# A cell-based infection assay identifies efflux pump modulators that reduce bacterial intracellular load

**DOI:** 10.1371/journal.ppat.1007115

**Published:** 2018-06-07

**Authors:** Abigail L. Reens, Amy L. Crooks, Chih-Chia Su, Toni A. Nagy, David L. Reens, Jessica D. Podoll, Madeline E. Edwards, Edward W. Yu, Corrella S. Detweiler

**Affiliations:** 1 Department of Molecular, Cellular, & Developmental Biology, University of Colorado Boulder, Boulder, CO, United States of America; 2 Department of Pharmacology, Case Western Reserve, Cleveland OH, United States of America; 3 Department of Physics, University of Colorado Boulder, Boulder, CO, United States of America; 4 JILA, National Institutes of Standards and Technology and University of Colorado Boulder, Boulder, CO, United States of America; University of Washington, UNITED STATES

## Abstract

Bacterial efflux pumps transport small molecules from the cytoplasm or periplasm outside the cell. Efflux pump activity is typically increased in multi-drug resistant (MDR) pathogens; chemicals that inhibit efflux pumps may have potential for antibiotic development. Using an in-cell screen, we identified three efflux pump modulators (EPMs) from a drug diversity library. The screening platform uses macrophages infected with the human Gram-negative pathogen *Salmonella enterica (Salmonella)* to identify small molecules that prevent bacterial replication or survival within the host environment. A secondary screen for hit compounds that increase the accumulation of an efflux pump substrate, Hoechst 33342, identified three small molecules with activity comparable to the known efflux pump inhibitor PAβN (Phe-Arg β-naphthylamide). The three putative EPMs demonstrated significant antibacterial activity against *Salmonella* within primary and cell culture macrophages and within a human epithelial cell line. Unlike traditional antibiotics, the three compounds did not inhibit bacterial growth in standard microbiological media. The three compounds prevented energy-dependent efflux pump activity in *Salmonella* and bound the AcrB subunit of the AcrAB-TolC efflux system with K_D_s in the micromolar range. Moreover, the EPMs display antibacterial synergy with antimicrobial peptides, a class of host innate immune defense molecules present in body fluids and cells. The EPMs also had synergistic activity with antibiotics exported by AcrAB-TolC in broth and in macrophages and inhibited efflux pump activity in MDR Gram-negative ESKAPE clinical isolates. Thus, an in-cell screening approach identified EPMs that synergize with innate immunity to kill bacteria and have potential for development as adjuvants to antibiotics.

## Author summary

Bacteria evolved molecular machines called efflux pumps to export toxic chemicals, including antibiotics encountered in the environment. Multi-drug resistant (MDR) bacteria use efflux pumps to rapidly transport clinical antibiotics out of the cell and thereby increase the dosage at which they tolerate antibiotics. One way to combat MDR pathogens may be to reduce the activity of efflux pumps and thereby increase pathogen sensitivity to existing antibiotics. We describe an infection-based screen that identified chemicals that inhibit bacterial efflux pump activity and show that these compounds bind to and block the activity of bacterial efflux pumps.

## Introduction

Human pathogens have become increasingly resistant to clinical antibiotics. Gram-negative bacterial pathogens are particularly problematic because their outer membranes are impermeable to many chemicals, and because many compounds that do enter the periplasm or cross the cellular membrane are immediately exported by efflux pumps. Multi-drug resistant (MDR) bacteria typically have increased gene copy number and/or production of efflux pumps, features demonstrated to contribute to the failure of clinical antibiotic treatment [1]. For these reasons, compounds that reduce efflux pump activity (efflux pump modulators, EPMs) are under investigation for their potential use in re-sensitizing MDR pathogens to existing antibiotics [2].

Three synthetic small molecules with EPM activity against Gram-negative bacterial pathogens have been well characterized. Phe-Arg β-naphthylamide (PAβN) was identified in a screen for compounds that increase the sensitivity of *Pseudomonas aeruginosa* to levofloxacin, an antibiotic and efflux pump substrate [3,4]. PAβN binds AcrB, the main component of the efflux system AcrAB-TolC, a member of the RND (resistance-nodulation-cell division) family of pumps. However, this compound was not developed as an antibiotic because medicinal chemistry could not separate EPM activity from unfavorable pharmacokinetics and toxicology, possibly reflecting off-target effects [5,6]. A second series of EPMs was identified in the same screen as PAβN. These pyridopyrimidines were subjected to medicinal chemistry, and the lead compound D13-9001 has efficacy against *Pseudomonas aeruginosa* during infection of rats [7,8]. Finally, a screen for chemicals that increase the sensitivity of *E*. *coli* to ciprofloxacin identified the pyranopyridine MBX2319 as an EPM that targets AcrB and has activity against multiple *Enterobacteriaceae* [9–12]. We identified three compounds that have activity as EPMs using a different approach—an in-cell screen for small molecules that prevent the replication of the Gram-negative pathogen *Salmonella enterica (Salmonella)* in mammalian cells.

## Results

### Screen for compounds that are antibacterial and non-toxic to mammalian cells

**Fig 1:**
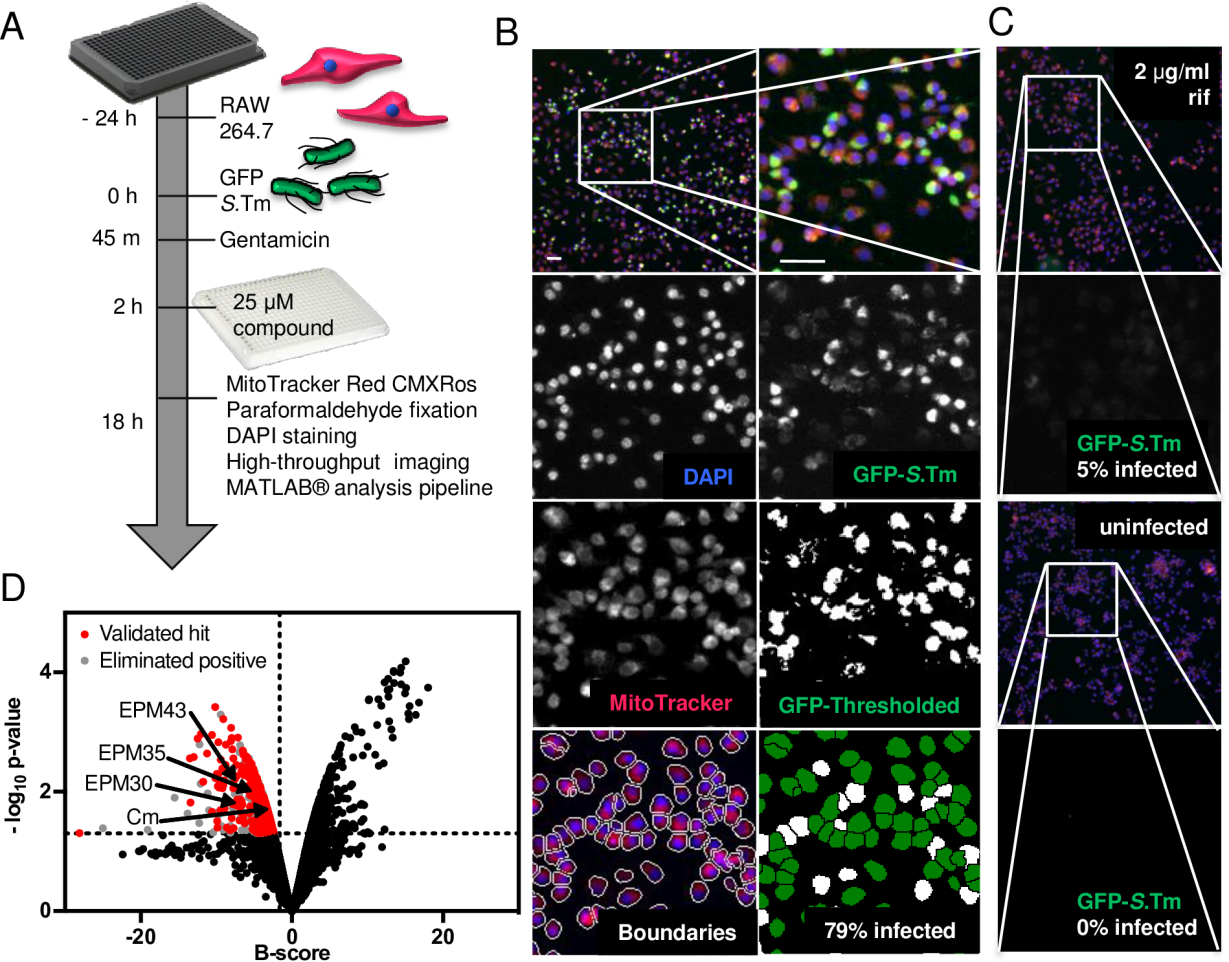
Screening platform (SAFIRE) used to identify EPMs. (A) Schematic of screening methodology. (B) Representative micrographs of infected macrophages from DMSO-treated wells. Upper left is a field with 522 macrophages; remaining images are the indicated channels zoomed in on the boxed region. Scale bars are 50 μm. (C) Representative micrographs of infected macrophages treated with rifampicin [2 μg/mL] or of uninfected macrophages treated with DMSO. (D) Distribution of B-scores and *p*-values for 14,400 compounds from the Maybridge HitFinder^TM^ v11 library. The locations of the three hit compounds (EPM30, EPM35 and EPM43) and of chloramphenicol (Cm), which was identified from the library, are shown.

SAFIRE *(**S**creen for* *A**nti-infectives using* *F**luorescence microscopy of* *I**ntracellula**R E**nterobacteriaceae)* is a high-content, medium-throughput screening platform that identifies compounds active against Gram-negative bacteria within the context of host cells (**Fig 1A**). The platform uses fluorescence microscopy and automated image analysis to monitor *Salmonella* within RAW 264.7 cells, a macrophage-like cell line in which the virulent *Salmonella* laboratory strain SL1344 replicates 10-15-fold [13–15].

We used SAFIRE to screen 14,400 compounds from a drug-like diversity library, the Maybridge HitFinder^TM^ v11 [16–19]. Macrophages in 384-well plates were infected with *Salmonella* expressing GFP under the control of a promoter, *sifB*, that is induced within macrophages (**Table 1**) [20]. After 45 minutes, infected macrophages were treated with the antibiotic gentamicin to prevent the replication of extracellular bacteria [21]. At two hours post-infection, test compounds [25 μM] were added. The compounds remained for the duration of the experiment. At 17.5 hours post-infection, when optimal *Salmonella* replication was observed, cells were stained with a marker of macrophage vitality, MitoTracker Red CMXRos, to help identify compounds toxic to eukaryotic cells. Thirty minutes later, cells were fixed and incubated with DAPI to stain DNA and imaged on an automated microscope. A MATLAB-based algorithm was used to quantify bacterial infection, specifically, the percentage of infected cells. Macrophage boundaries were first established using MitoTracker and DAPI signals (**Fig 1B)**. The percentage of infected cells was determined by setting a threshold for the GFP signal based on infected and uninfected controls; cells containing at least two GFP-positive pixels were labeled infected. The library was screened in duplicate, and well-to-well variability was addressed using B-score normalization [22–24]. Assay positives were called based on an activity threshold greater than one standard deviation below the mean B-score, and a *p*-value of less than 0.05 calculated using a modified t-test assuming an inverse gamma distribution of variances (**Fig 1D**) [23,25]. The micrographs of the 461 assay positives were manually reviewed to eliminate host-toxic and/or autofluorescent chemicals. The remaining 309 hits were retested using SAFIRE in the 96-well format and ranked based on reduction of bacterial load as determined by SAFIRE and traditional lysis and plating for colony forming units (CFU) (**S1 Table**). Sixty-four (85%) of the top 75 compounds, including chloramphenicol, reduced CFU by at least 25% (**S1 Fig**). The top 60 compounds (excluding chloramphenicol) were repurchased and validated using SAFIRE in a 96-well format; 58 repurchased compounds were active.

**Table 1:** Bacterial strains.

Reference #	Species	Strain name	Antibiotic^2^	Source
CSD1	*S*. *enterica*^1^	WT^3^, SL1344	Str	[26]
CSD1021	*S*. *enterica*^1^	*sifB*::GFP	Str, Kan	[20] SDFR3
CSD222	*S*. *enterica*^1^	*rpsM*::GFP	Str	[27] SM022
ALR1268	*E*. *coli*	RAM121		[28]
ALR1228	*S*. *enterica*^1^	mTagBFP2^4^	Str, Amp	[29]
ALR1257	*S*. *enterica*^1^	*acrAB*::kan	Str, Kan	This study
ALR1258	*S*. *enterica*^1^	*macAB*::kan	Str, Kan	This study
ALR1248	*S*. *enterica*^1^	S10801	Str, Amp, Tet	BEI Resources (NR-22067)
ALC1250	*E*. *coli*	1101362	Mero	BEI Resources (BAA-2340)
ALC1253	*K*. *pneumoniae*	1101160	Mero	BEI Resources (BAA-2342)
ALC1251	*K*. *pneumoniae*	ART 2008133	Mero	BEI Resources (BAA-1705)
ALC1252	*Enterobacter cloacae*	1101152	Mero	BEI Resources (BAA-2341)

^1^*S*. *enterica* are serovar Typhimurium. All but S10801 are in an SL1344 background.

^2^ Antibiotics for growth selection: Streptomycin (Str), Ampicillin (Amp), Kanamycin (Kan), Tetracycline (Tet), Meropenem (Mero)

^3^ Wild-Type (WT)

^4^ mTagBFP2 is on pACYC177

### Three compounds may modulate bacterial efflux pump activity

**Fig 2:**
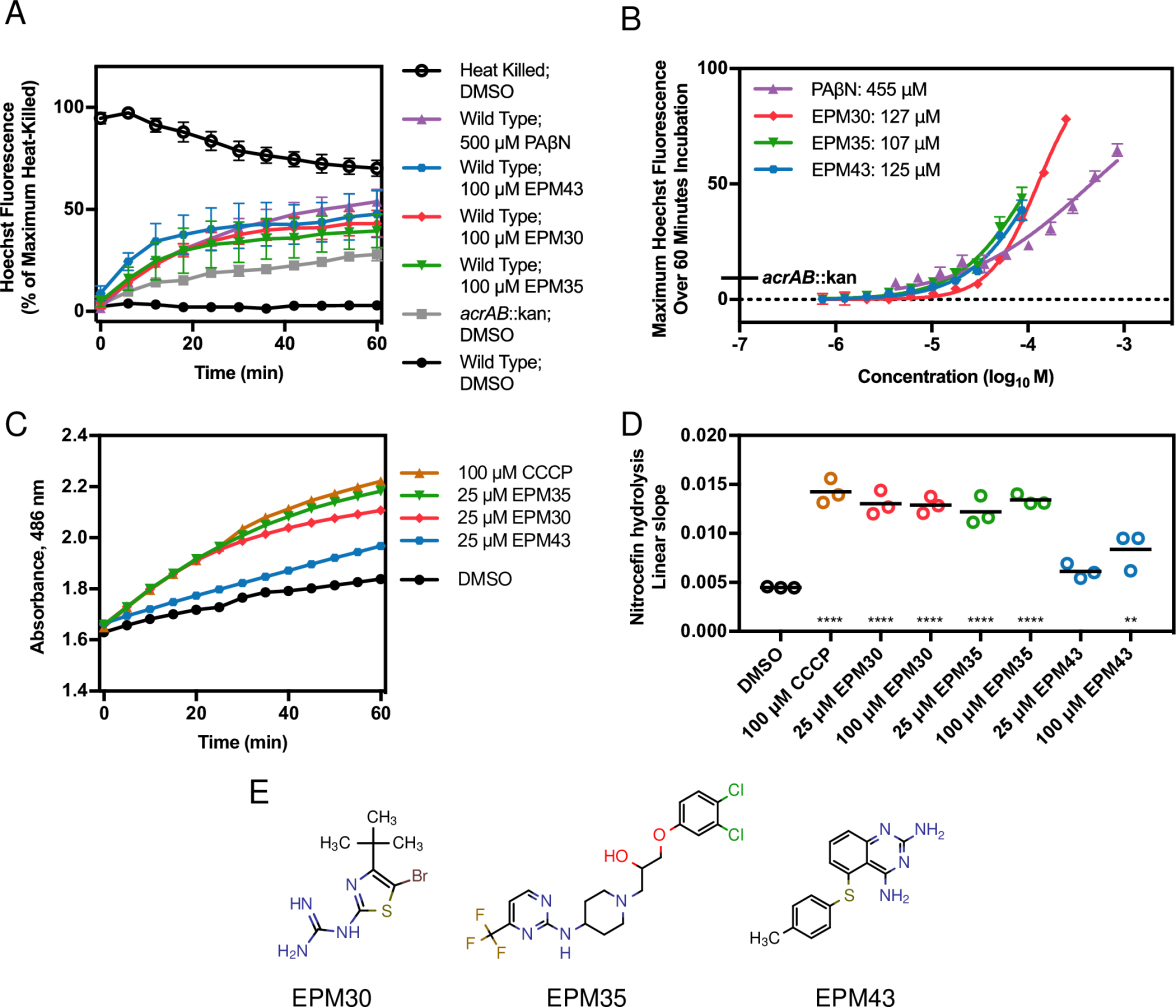
Three compounds may modulate bacterial efflux pump activity. (A, B) *Salmonella* was incubated with Hoechst 33342 and the indicated compound. (A) Fluorescence was normalized to the maximum of heat-killed bacteria (100%). Mean and SEM from three biological replicates. (B) Maximum fluorescence over 60 minutes of exposure normalized to the maximum fluorescence of heat-killed bacteria (100%). The EC_50_s in the key were established using a nonlinear four-parameter fit. Mean and SEM from three biological replicates, each performed in duplicate. (C, D) Nitrocefin [100 μM] hydrolysis in a membrane-compromised *E*. *coli* strain treated with the indicated concentrations of drugs. Hydrolysis increases when efflux is blocked. (C) Absorbance 486 nm of RAM121 *E*. *coli*. Mean and SD are representative of three independent biological replicates. (D) Slope of the linear region of the A_486_ plot from at least three experiments. Data is normalized to A_486_/minute. ** *p* < 0.001, **** *p* < 0.0001 by one-way ANOVA with Dunnett’s post-test. (E) Structures of the three EPMs.

SAFIRE has the potential to identify EPMs because *Salmonella* requires at least two efflux pumps, AcrAB and MacAB, to replicate and/or survive within macrophages and mice [30–34]. The fluorescent dye Hoechst 33342 is an efflux pump substrate, and increased Hoechst accumulation relative to controls identifies potential modulators of efflux pumps [35]. We incubated bacteria with each of the 58 repurchased, validated hits and Hoechst 33342. As expected, heat-killed bacteria exhibited high fluorescence immediately after exposure to Hoechst because an electrochemical gradient is required to export pump substrates. Live, wild-type *Salmonella* demonstrated low fluorescence, and a strain lacking the AcrAB efflux pump had a modest level of fluorescence. PAβN treatment resulted in higher levels of fluorescence, as expected [35]. Under the same conditions, treatment with three of the 58 compounds (EPM30, EPM35 and EPM43) resulted in fluorescence comparable to that of PAβN (**Fig 2A**). Further examination revealed that the three compounds had half maximum effective concentrations (EC_50_s) four-fold lower than that of PAβN in the Hoechst assay (**Fig 2B**). These observations show that the hit compounds increase bacterial accumulation of an efflux pump substrate.

We next tested the ability of the three hit compounds to inhibit efflux of nitrocefin, a chromogenic beta-lactam and known AcrAB substrate [9,36]. *E*. *coli* strain RAM121 encodes a porin with a large diameter, which allows rapid influx of nitrocefin, and the periplasmic AmpC beta-lactamase, which hydrolyzes nitrocefin and results in a color change. Carbonyl cyanide m-chlorophenylhydrazone (CCCP) is a protonophore that inhibits efflux pumps, causing increased nitrocefin hydrolysis (**Fig 2C and 2D**). Treatment with EPM30 or EPM35 yielded a similar result, whereas treatment with EPM43 only modestly increased hydrolysis. Thus, the three hit compounds (**Fig 2E**) may inhibit bacterial efflux pumps.

### The three hit compounds are antibacterial against *Salmonella* in macrophages and epithelial cells

**Fig 3:**
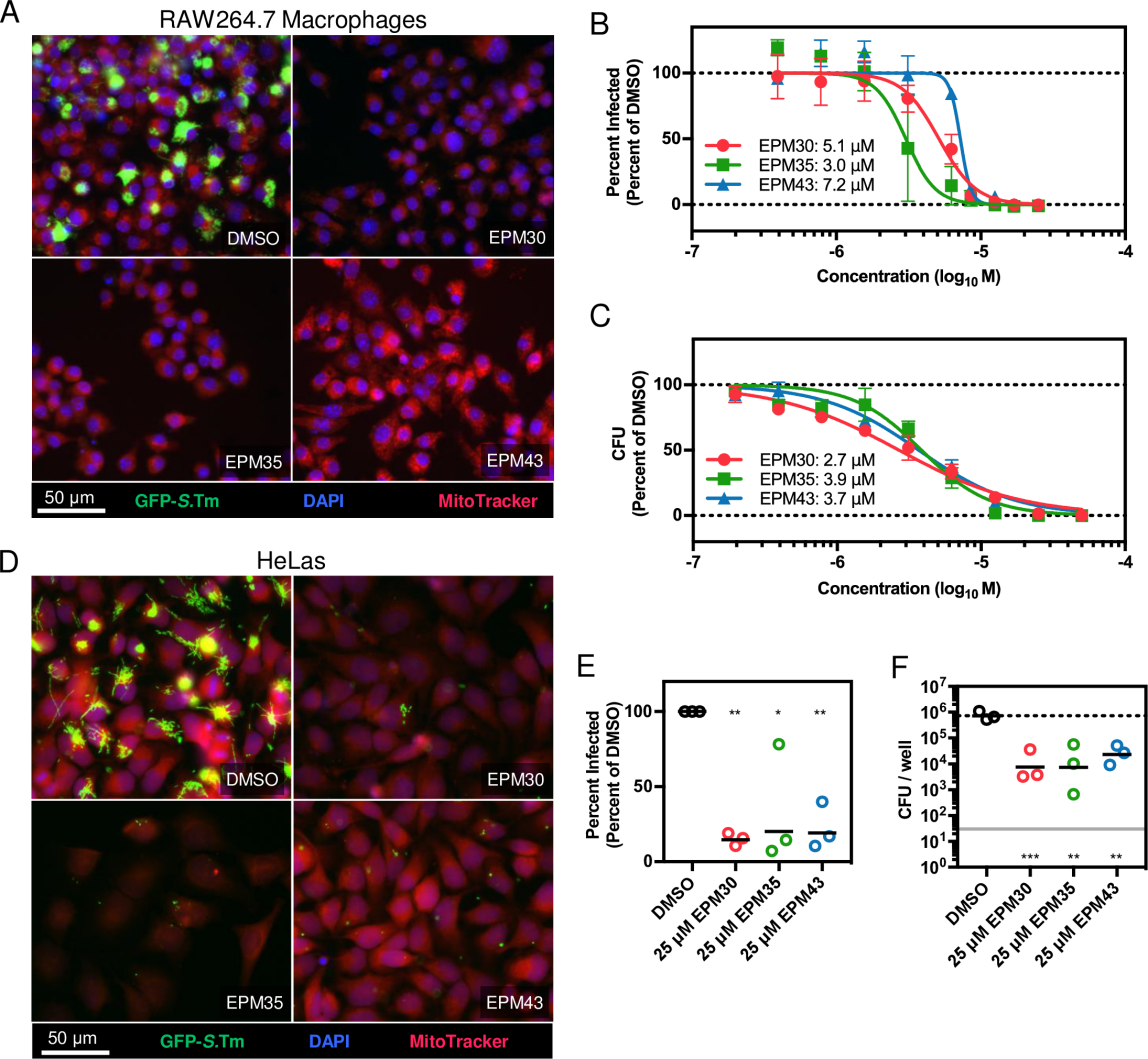
The three hit compounds decrease bacterial load of *Salmonella* in mammalian cells. (A-C) Monitoring of bacterial load by GFP (SAFIRE) or CFU in RAW264.7 cells. (A) Representative micrographs of cells in 96-well plates infected with *sifB*::*GFP Salmonella*. Two hours after infection cells were treated with the indicated compound [25 μM] for 16 hours. (B) Dose response curve for SAFIRE and (C) CFU; keys includes IC_50_ values. (D-E) Monitoring of bacterial load by GFP (SAFIRE) in HeLa cells infected with *Salmonella* expressing *rpsM*::*GFP* and treated with the indicated compound [25 μM]. (F) Monitoring of bacterial load by CFU in BMDMs treated with compounds [25 μM]. Mean and SEM from three independent biological replicates. The nonlinear curve fitting (B, C) is constrained using uninfected cells as the minimum and DMSO-treated cells as the maximum. (E, F) * *p* < 0.05; ** *p* < 0.01, *** *p* < 0.001 compared to DMSO by one-way ANOVA with Dunnett’s post-test.

We performed a more thorough characterization of the putative EPMs regarding anti-*Salmonella* activity in multiple mammalian cell types. Micrographs from RAW264.7 macrophages treated with 25 μM of each compound demonstrated a significant reduction in the percentage of GFP-positive cells compared to treatment with vehicle alone (**Fig 3A**). The SAFIRE inhibitory concentration-50 (IC_50_) for the three compounds in macrophages ranged from 3 to 7 μM (**Fig 3B**). To establish whether reduced GFP signal correlates with bacterial killing, we quantified bacterial survival by enumerating CFU from infected cells. The IC50s for the 3 compounds by CFU ranged from 2 to 5 μM (**Fig 3C**). HeLa cells harboring a *Salmonella*-GFP expressing strain and treated with 25 μM of each compound also demonstrated a reduction in the percentage of GFP-positive cells compared to treatment with vehicle alone (**Fig 3D and 3E**). In primary bone marrow-derived mouse macrophages (BMDMs) all three hit compounds reduced the number of recoverable *Salmonella* by approximately 20-fold (**Fig 3F**). Finally, while the macrophage and HeLa cell culture assays require gentamicin to prevent the replication of extracellular *Salmonella*, we established in broth assays that the three compounds do not synergize with the antibacterial activity of gentamicin (**S2 Fig**). Thus, the putative EPMs inhibit bacterial replication and/or survival in at least two cell types, macrophages and epithelial cells, which are relevant to whole animal infection.

### The three hit compounds reduce the survival of MDR *Salmonella* in macrophages

**Fig 4:**
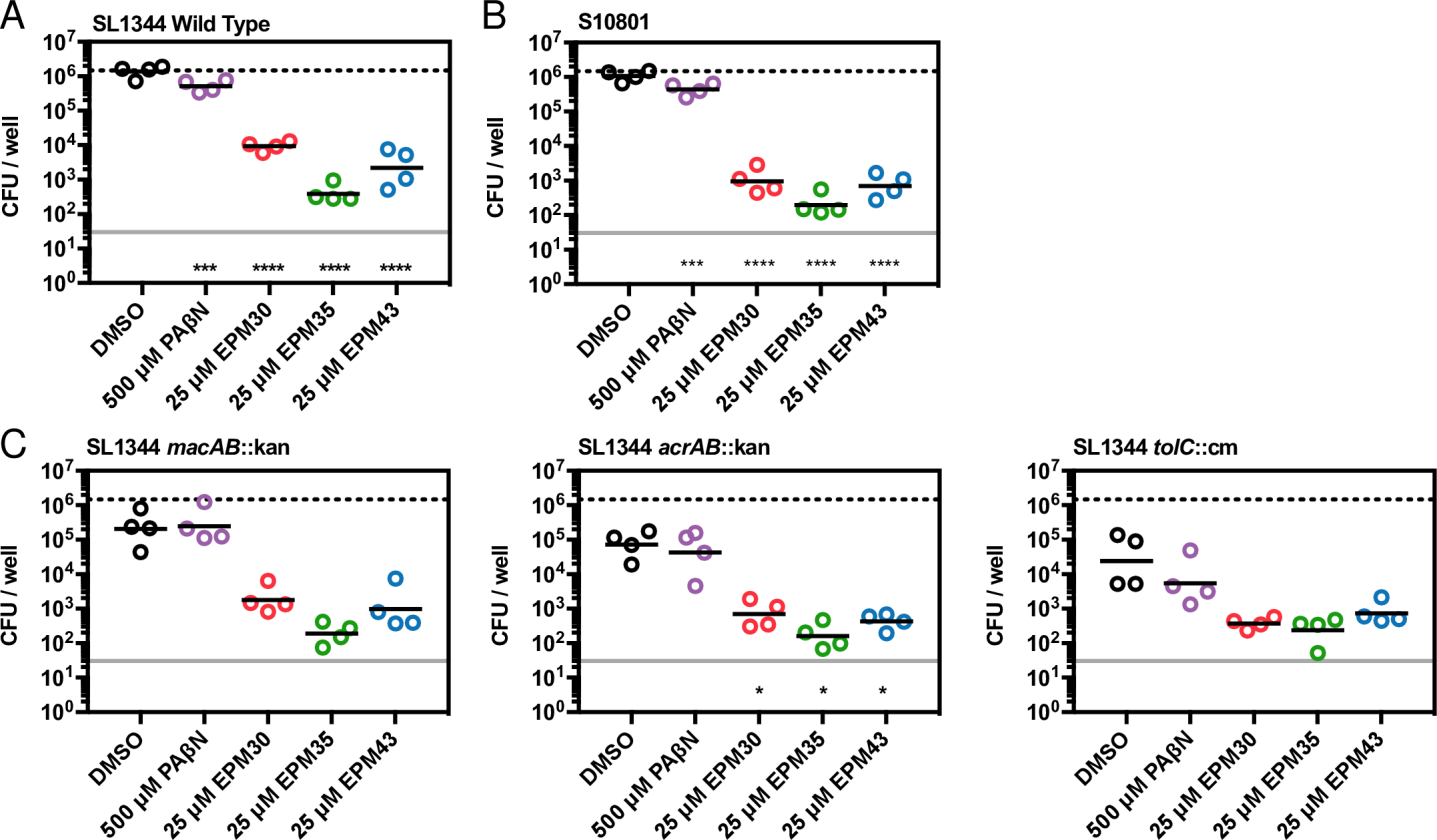
The hit compounds reduce the survival of MDR *Salmonella* in macrophages. Monitoring of bacterial load by CFU in RAW264.7 cells infected for two hours with the strain of *Salmonella* shown and then treated with the indicated compound for 16 hours, followed by macrophage lysis and plating for CFU. A) SL1344 Wild Type *Salmonella*, B) Clinical *Salmonella* isolate S10801, (C) SL1344 with *macAB*::kan, *acrAB*::kan or *tolC*::cm, respectively. Geometric mean of four biological replicates. Upper lines, mean CFU/well of wild-type SL1344 with DMSO treatment; lower lines, limit of detection. * *p* < 0.05, ** *p* < 0.01; *** *p* < 0.001, **** *p* < 0.0001 relative to DMSO, one-way ANOVA with Dunnett’s post-test.

We next examined whether the bacterial load of MDR *Salmonella* in macrophages is reduced upon treatment of infected cells with the hit compounds. A clinical MDR *Salmonella* isolate (S10801) was recovered from hit compound-treated macrophages at levels 1000-fold lower than from DMSO-treated macrophages (**Fig 4**). These results indicate that the three compounds inhibit not only SL1344 but also an MDR clinical isolate during infection of cells.

Clinical MDR isolates frequently express high levels of efflux pumps [1]. The two *Salmonella* efflux pumps needed for bacterial survival in cells and mice, AcrAB and MacAB, both use the TolC channel to export cargo across the outer membrane [30–34,37]. We first confirmed that *Salmonella* strains lacking *acrAB*, *macAB*, or *tolC* replicate poorly in macrophages compared to the wild-type parent strain (**Fig 4**). Treatment of macrophages with any of the three compounds reduced loads of wild-type bacterial below those observed in DMSO-treated cells infected with the *acrAB*, *macAB*, or *tolC* mutant strains. Compound treatment of the mutant strains in macrophages further reduced the levels of mutant bacteria. Given that *Salmonella* encodes other efflux pumps that may contribute to survival in macrophages in the absence of *macAB*, *acrAB*, or *tolC* [34,38,39], these data could suggest that the hit compounds target other efflux pumps.

### The hit compounds inhibit energy-dependent efflux pump activity

Having established that the hit compounds are antimicrobial in mammalian cells, we returned to the analysis of their activity. While the Hoechst accumulation assay is a good first approximation of anti-efflux pump activity, quantification of export in real time based on energy (glucose) dependence is a more specific assay for pump inhibition.

**Fig 5:**
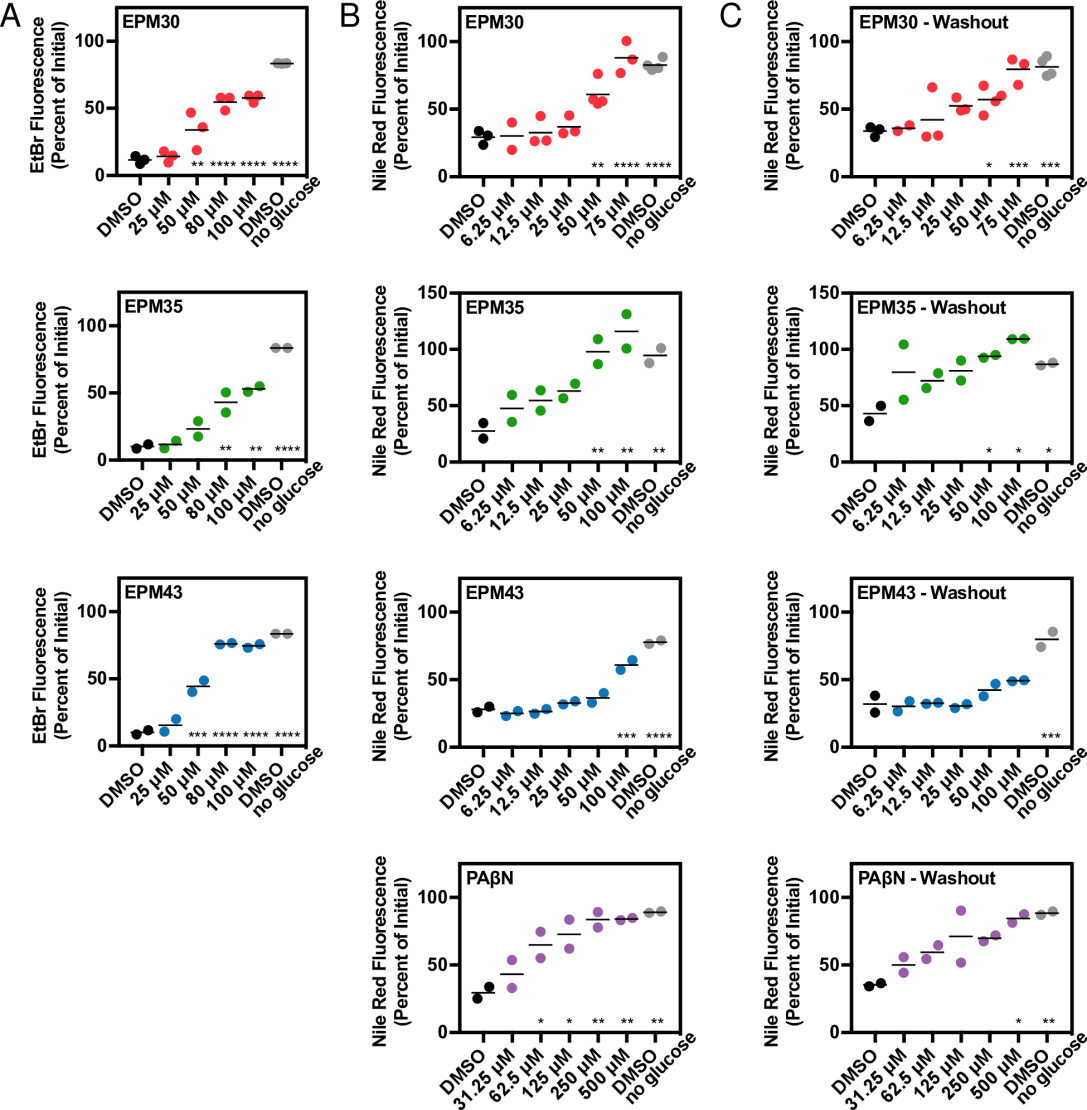
EPMs block efflux of ethidium bromide and Nile red. *Salmonella* were incubated with either ethidium bromide or Nile red without glucose, treated with compound, exposed to glucose, and then monitored for fluorescence. Data for each sample were normalized to the initial fluorescence (100%). Dose response curves are (A) ethidium bromide at 28 minutes after glucose addition, (B) Nile red at 7 minutes after glucose addition, and (C) Nile red after washout of compound 7 minutes after glucose addition. Mean of at least two biological replicates performed in duplicate. * *p* < 0.05; ** *p* < 0.01; *** *p* < 0.001; ****p < 0.0001 compared to DMSO + glucose by one-way ANOVA and Dunnett’s multiple comparison post-test.

Ethidium bromide is an efflux pump substrate that fluoresces upon intercalating into DNA. We pre-loaded cells with ethidium bromide and then treated with glucose to energize the efflux pumps and stimulate export [40]. Incubation with PAβN or any of the three putative EPMs reduced ethidium bromide export upon glucose addition in a dose-dependent manner (**Fig 5A; S3A Fig**). A similar assay using the efflux pump substrate Nile red further demonstrated that EPM30 and EPM35 reduced pump export (**Fig 5B; S3B Fig; S4 Fig**). Nile red becomes strongly fluorescent upon partitioning into the cytoplasmic membrane and possibly the inner leaflet of the outer membrane but is rapidly exported [41–44]. Washout of the EPMs still reduced Nile red export, suggesting the activity of these compounds is not readily reversible (**Fig 5C; S3C Fig**). These observations indicate that the three hit compounds may inhibit energy-dependent efflux pump activity.

### Bacterial membranes remain intact upon exposure to the three EPMs

**Fig 6:**
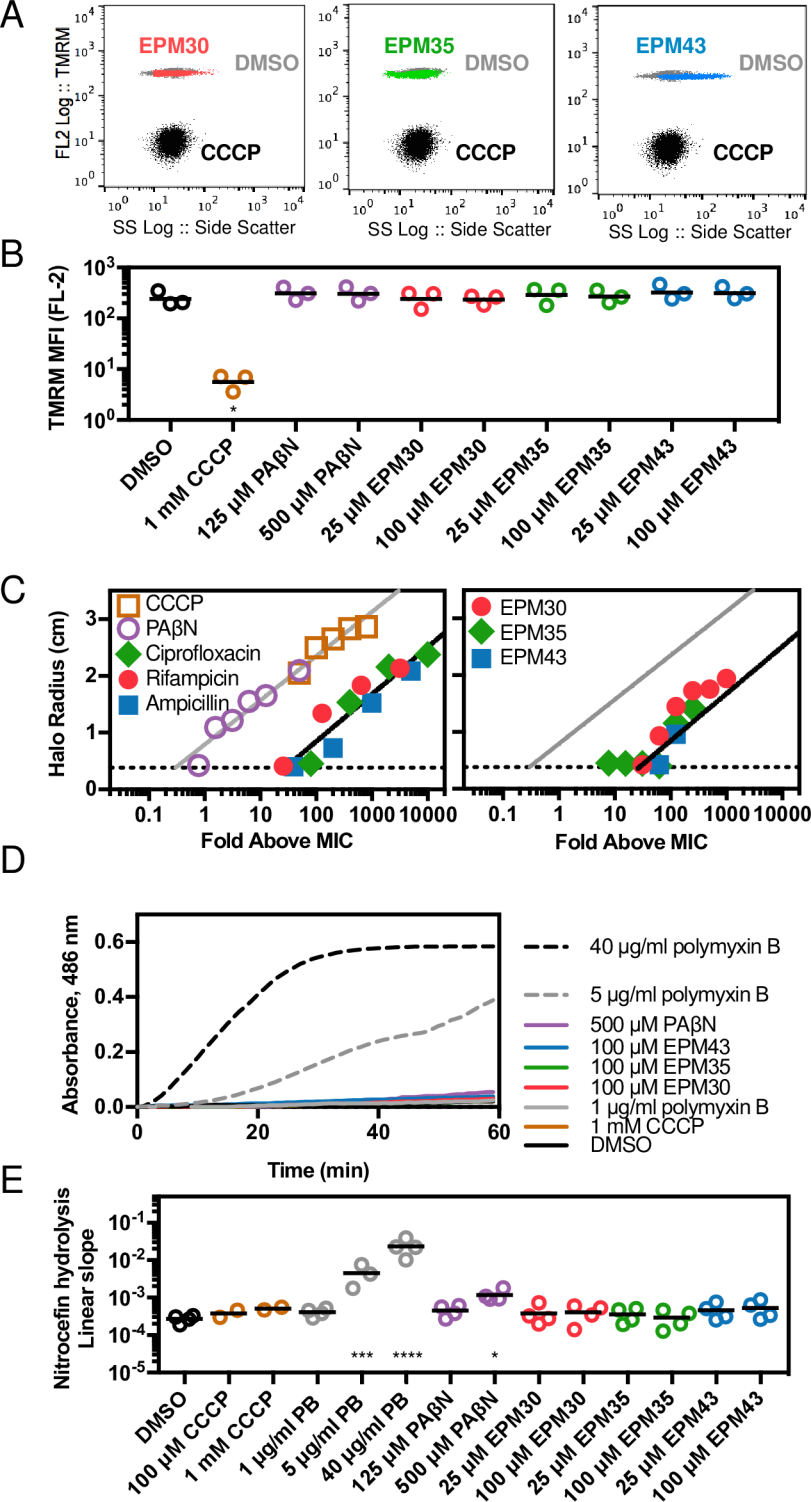
EPMs do not disrupt bacterial inner or outer membranes. (A,B) *Salmonella* treated with DMSO or EPMs [100 μM] but not CCCP [1 mM] acquire TMRM staining within 30 minutes. (A) Representative data from one of three independent experiments. (B) Median fluorescence intensity from three experiments normalized to unstained control (0). (C) Disk diffusion assays; the radius of the zone of growth inhibition after 16 hours of exposure to compound across a dose range. Black lines, semilog fit for the combined antibiotic data; gray lines, semilog fit for CCCP and PAβN; dotted lines, limit of detection (disk radius). Average of two measurements from each image captured from one experiment representative of two independent experiments. (D,E) Nitrocefin access to the periplasm as monitored by nitrocefin [100 μM] hydrolysis in the presence of the indicated concentrations of compounds. (D) Absorbance 486 nm of *bla+ Salmonella* normalized to *bla- Salmonella*. Data is representative of at least two independent biological replicates. (E) Slope of the linear region of the A_486_ plot from at least three experiments. Data is normalized to A_486_/minute. * *p* < 0.05, *** *p* < 0.001, **** *p* < 0.0001 by one-way ANOVA with Dunnett’s post-test.

Since efflux pumps rely upon the proton motive force or ATP to provide the energy for the transport of substrates, chemicals that disrupt the inner membrane may indirectly inhibit efflux. To establish whether the three EPMs alter bacterial inner membrane potential, we observed their effect on the incorporation of the voltage-sensitive dye tetramethylrhodamine methyl ester (TMRM). After 30 minutes of exposure to the ionophore CCCP, TMRM levels in cells were approximately 50-fold lower than upon treatment with DMSO, but treatment with any of the three EPMs did not alter TMRM signal based on analyses by flow cytometry (**Fig 6A and 6B**). These observations suggest that membrane potential remains intact in the presence of the EPMs. To establish whether a longer incubation with the EPMs may compromise membrane integrity, we monitored the effect of the EPMs on bacterial swimming, an energy intensive activity, over 15 hours [45] (**Fig 6C; S5 Fig**). *Salmonella* were injected into the center of soft-agar plates and 10 μl of compound was pipetted onto paper disks on the periphery [45]. Control compounds included CCCP and PAβN, which disrupts membranes over long (> 30 minutes) exposures [46–48]. Since the swimming assay also requires bacterial growth, we tested whether filters containing bacteriostatic antibiotics that are not known to disrupt membranes prevented swimming. Neither the three EPMs nor the conventional antibiotics inhibited swimming relative to their MIC, as compared to CCCP and PAβN, further suggesting that the EPMs do not interfere with bacterial energy production across the inner membrane.

A second class of chemicals that appears to interfere with bacterial efflux does so by permeabilizing the outer membrane, which allows substrates to diffuse into the periplasm [36]. We therefore tested whether exposure of bacteria with an intact outer membrane to the EPMs would enable the beta-lactam nitrocefin to enter the periplasm, as monitored by nitrocefin hydrolysis in bacteria with high expression levels of *bla* beta-lactamase [49]. In this assay, monitoring of absorbance showed that CCCP had no effect on nitrocefin hydrolysis, whereas polymyxin B, a pore-forming antimicrobial peptide, increased hydrolysis by 10-fold or more. The efflux pump inhibitor PAβN slightly increased hydrolysis but the EPMs did not (**Fig 6D and 6E**). Thus, the EPMs did not appear to increase nitrocefin access to the periplasm, suggesting they do not increase bacterial outer membrane permeability.

### The three EPMs bind the efflux pump AcrB

**Fig 7:**
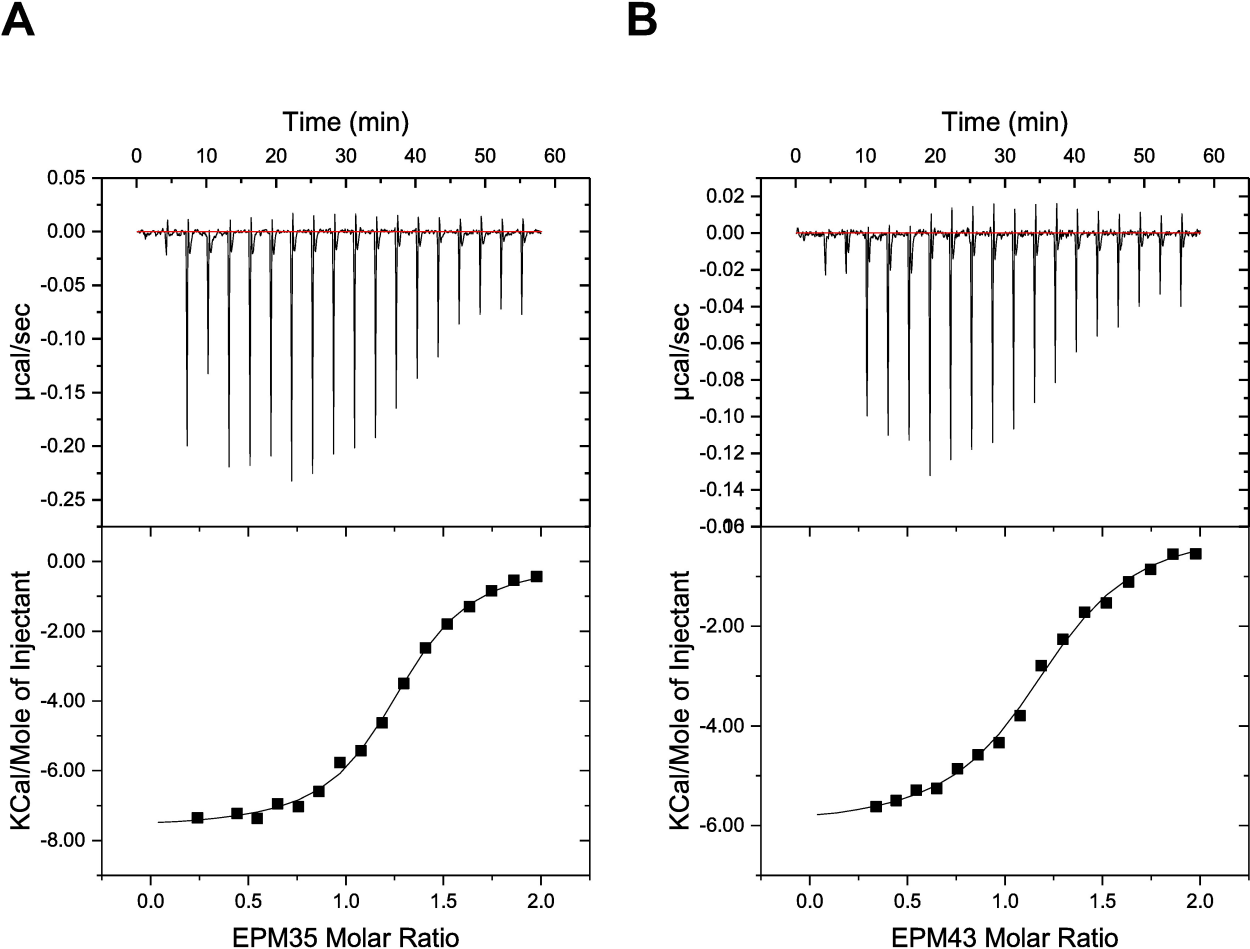
EPM35 and EPM43 interact with the efflux pump AcrB. (A, B) Representative ITC for the binding of (A) EPM35 and (B) EPM43 to *E*. *coli* AcrB. Each peak in the upper graphs corresponds to the injection of 2 μL of 100 μM of the EPM in buffer containing 20 mM Na-HEPES (pH 7.5), 0.05% DDM and 5% DMSO into the reaction containing 10 μM of *E*. *coli* monomeric AcrB in buffer containing 20 mM Na-HEPES (pH 7.5), 0.05% DDM and 5% DMSO. The lower graphs show the cumulative heat of reaction displayed as a function of injection number. The solid line is the least-square fit to the experimental data.

Since all three EPMs reduced Nile red or ethidium bromide export in *Salmonella*, well-studied substrates of the AcrAB-TolC efflux system [40,50], we established whether any of the compounds bind *E*. *coli* AcrB using isothermal titration calorimetry (ITC) [51–53] (**Fig 7; S6 Fig; Table 2**). The equilibrium dissociation constants (K_D_s) are within the micromolar range, demonstrating that AcrB is capable of binding each of the EPMs. It appears that AcrB interacts with these three EPMs using different binding mechanisms. The free energies of binding for EPM35 and EPM43 are dominated by the large negative enthalpies (ΔHs) of -7.7 and -6.0 kcal/mol, respectively. However, the binding for EPM30 is predominantly determined by its entropic contribution (-TΔS), which is -6.8 kcal/mol at 25 ^o^C. These data suggest that the AcrB efflux pump tends to bind the EPMs with a 1:1 protein monomer-to-ligand molar ratio.

**Table 2:** Binding of inhibitors by AcrB.

	*K*_*D*_ (μM)	*ΔH* (cal•mol^-1^)	*ΔS* (cal•mol•deg^-1^)	N
EPM30	1.79 ± 0.37	-1066.0 ± 46.5	22.7	1.3 ± 0.05
EPM35	0.29 ± 0.03	-7663.1 ± 84.4	4.2	1.2 ± 0.01
EPM43	0.56 ± 0.06	-6033.2 ± 106.3	8.6	1.2 ± 0.01

### The hit compounds are not antibacterial in standard bacterial medium

To establish whether the hit compounds have minimum inhibitory concentrations (MICs) in broth that are similar to their IC_50_s in host cells (**Fig 3B and 3C**), we examined bacterial growth in their presence using standard rich laboratory media, Mueller Hinton Broth (MHB). The MICs of EPM30, EPM35, and EPM43 respectively were 100 μM [36 μg/mL], 400 μM [186 μg/mL], and >400 μM [113 μg/mL], for two *Salmonella* strains: the strain used in the SAFIRE screen (SL1344), and a clinical MDR *Salmonella* isolate (S10801). These broth MIC values (> 100 μM) are considerably higher than the IC_50_s observed in host cells (< 10 μM). Thus, the three putative EPMs may not function like traditional antibiotics and yet are potent in the context of the host cell.

### The three EPMs sensitize bacteria to antimicrobial peptides

**Fig 8:**
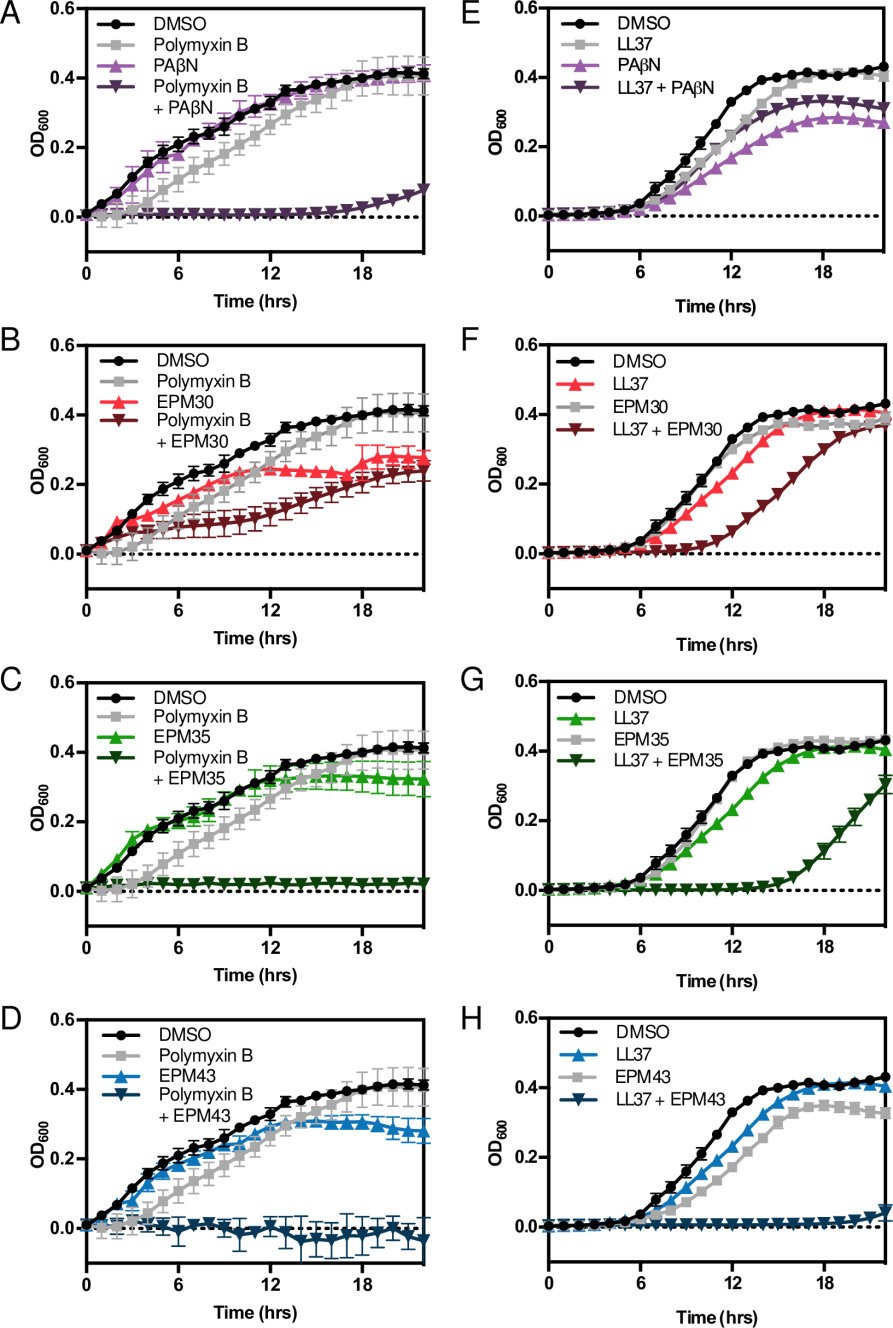
EPMs synergize with antimicrobial peptides. *Salmonella* was grown in M9-based defined media in the presence of **(A-D**) polymyxin B (5 μg/ml; 1/8 MIC) or **(E-H)** LL37 (5 μg/ml; 1/8 MIC) and EPMs (PAβN, 500 μM; EPMs, 25 μM). Mean and SD of triplicate samples from one representative experiment of three independent biological replicates. DMSO, polymyxin B and LL37 curves repeat across graphs.

We next addressed why the EPMs kill bacteria in mammalian cells at concentrations 10-fold lower than they inhibit efflux in broth. One possibility is that the presence of antimicrobial peptides (AMPs) within host cells plays a role. Mammalian cells constitutively express AMPs and increase AMP expression in response to infection [54,55]. We found that in broth the combination of each EPM with either the bacterial-derived polymyxin B or the human cathelicidin AMP LL37, but not individual treatments, significantly inhibited *Salmonella* growth; EPM35 and EPM43 display stronger synergy than EPM30 in this regard (**Fig 8**). These data suggest at least three possibilities: AMPs may potentiate EPM activity, EPMs may potentiate AMP activity, or both.

**Fig 9:**
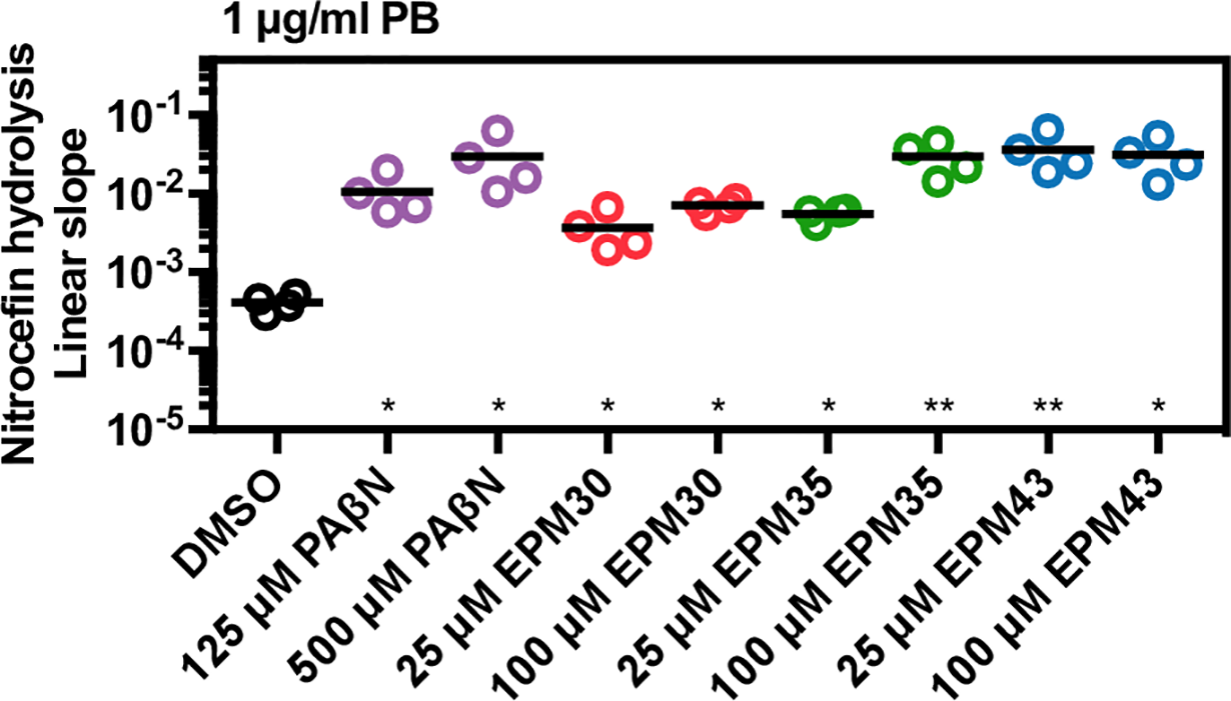
EPMs sensitize bacteria to outer membrane permeabilization by antimicrobial peptides. Nitrocefin access to the periplasm in the presence of a non-permeabilizing concentration of polymyxin B [1 μg/ml] and the indicated concentrations of EPMs, quantitated as in Fig 6E. * *p* < 0.05, ** *p* < 0.01 by one-way ANOVA with Dunnett’s post-test.

To distinguish between these possibilities, we first determined that bacterial exposure to polymyxin B concentrations high enough to allow nitrocefin access to the periplasm (5 μg/mL, **Fig 6D and 6E**) did not enhance the ability of the EPMs to increase Hoechst accumulation (**S7A Fig**). Similarly, co-treatment of polymyxin B with EPMs did not synergistically increase Nile red retention compared to polymyxin B or EPMs alone (**S7B Fig**). These observations suggest that the membrane-damaging activity of polymyxin B did not potentiate EPM blockage of efflux pumps. We next observed that low concentrations of polymyxin B [1 μg/mL], which do not by themselves allow nitrocefin access to the periplasm (**Fig 6D and 6E**), increased the rate of nitrocefin hydrolysis in a *bla*-expressing *Salmonella* strain when EPMs were present [25 μM] (**Fig 9**). It thus appears that EPMs potentiate AMPs with regard to both nitrocefin entry into the periplasm and bacterial growth inhibition. Therefore, EPMs may decrease the effective concentration of AMPs and, for this reason, have indirect antibacterial activity in the context of the host.

### Two EPMs act synergistically with known AcrAB substrates in broth

We next established whether co-incubation with EPMs reduced the MIC of established AcrB substrates in the wild-type SL1344 strain and the S10801 clinical isolate. Exposure to EPM35 or EPM43 decreased by four-fold the MIC of chloramphenicol for one or both strains (**Table 3**). EPM35 was also synergistic with tetracycline and erythromycin. Similar effects were observed in the SL1344-derived *macAB* mutant strain but not in the *acrAB* or *tolC* mutant strains. These data suggest that the AcrAB-TolC efflux pump may be a relevant target for EPM35 and EPM43 with regard to antibiotic potentiation, and more importantly, that these EPMs appear to reduce the effective dose in broth of some clinical antibiotics.

**Table 3:** MICs (μg/mL) of AcrB substrates in combination with EPMs.

		SL1344	S10801	*macAB*::kan	*acrAB*::kan	*tolC*::cm
Tetracycline	DMSO	2	128	2	0.5	0.5
	25 μM EPM30	2	128	2	0.5	0.5
	25 μM EPM35	0.5	64	0.5	0.5	0.5
	25 μM EPM43	1	64	1	0.5	0.5
Chloramphenicol	DMSO	4	256	4	2	
	25 μM EPM30	2	256	2	2	
	25 μM EPM35	1	64	1	1	
	25 μM EPM43	1	128	1	1	
Ciprofloxacin	DMSO	0.004	0.016	0.004	0.002	0.002
	25 μM EPM30	0.002	0.008	0.002	0.002	0.002
	25 μM EPM35	0.002	0.008	0.002	0.002	0.002
	25 μM EPM43	0.002	0.008	0.002	0.002	0.002
Novobiocin	DMSO	512	512	512	256	256
	25 μM EPM30	256	256	256	256	256
	25 μM EPM35	256	256	256	256	256
	25 μM EPM43	256	256	256	256	256
Erythromycin	DMSO	128	64	128	32	32
	25 μM EPM30	64	64	64	32	32
	25 μM EPM35	32	32	32	32	32
	25 μM EPM43	64	32	64	32	32
Crystal Violet	DMSO	16	16	16	8	8
	25 μM EPM30	16	16	16	8	8
	25 μM EPM35	8	8	8	8	8
	25 μM EPM43	8	8	8	8	8

### The EPMs act synergistically with erythromycin or ciprofloxacin in macrophages

**Fig 10:**
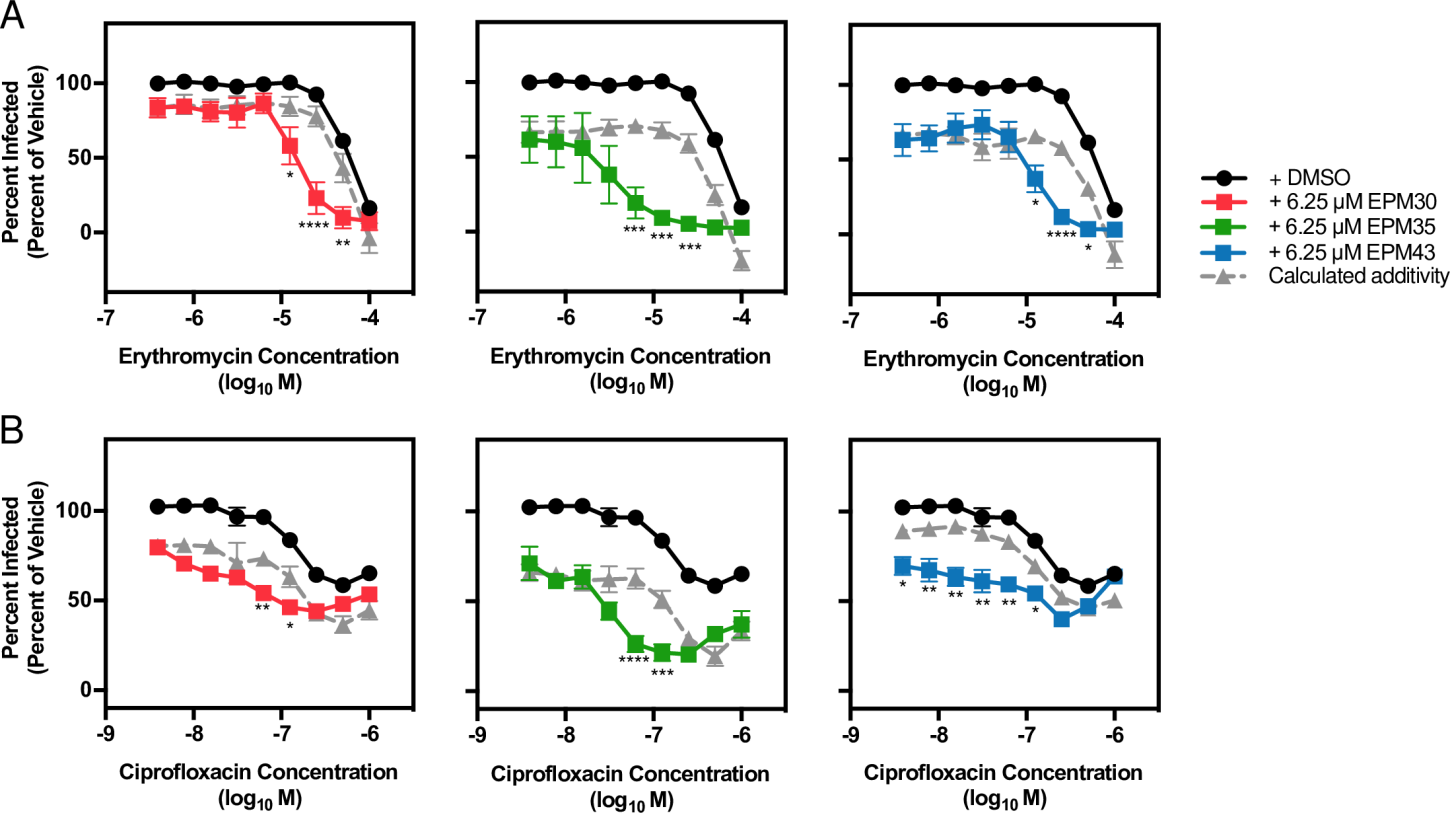
EPMs enhance activity of erythromycin and ciprofloxacin against *Salmonella* in macrophages. RAW 264.7 macrophages were infected with *Salmonella* and treated with a dose range of (A) erythromycin or (B) ciprofloxacin and with DMSO or the indicated concentration of an EPM. At 18 hours post infection samples were processed for fluorescence microscopy as described. Data were normalized to treatment with DMSO and antibiotic vehicle (100%). Key: black, DMSO; red, EPM30; green, EPM35; blue, EPM43; gray, calculated additivity of the antibiotic and the corresponding EPM using the formula (100 –([percent inhibition EPM] + [percent inhibition antibiotic])), where percent inhibition is calculated as 100 –[percent of DMSO]. Data are mean + SEM of three biological replicates. * *p* < 0.05; ** *p* < 0.01; *** *p* < 0.001; **** *p* < 0.0001 of EPM treatment versus calculated additivity by one-way ANOVA with Sidak’s multiple comparison test.

Since intracellular conditions are distinct from microbiological media, we tested whether the EPMs also synergize with AcrAB-exported antibiotics for bacterial killing in macrophages. RAW264.7 macrophages were infected with wild type *Salmonella* followed two hours later by treatment with antibiotic over a dose range with or without an EPM [6.25 μM]. SAFIRE analysis revealed that both erythromycin and ciprofloxacin were potentiated by the EPMs, as indicated by a difference between the co-treatment data and calculated additivity curves (**Fig 10**). Thus, the EPMs may reduce the effective dose of erythromycin and ciprofloxacin in macrophages.

### EPM35 and EPM43 have anti-efflux activity in MDR ESKAPE pathogens

**Fig 11:**
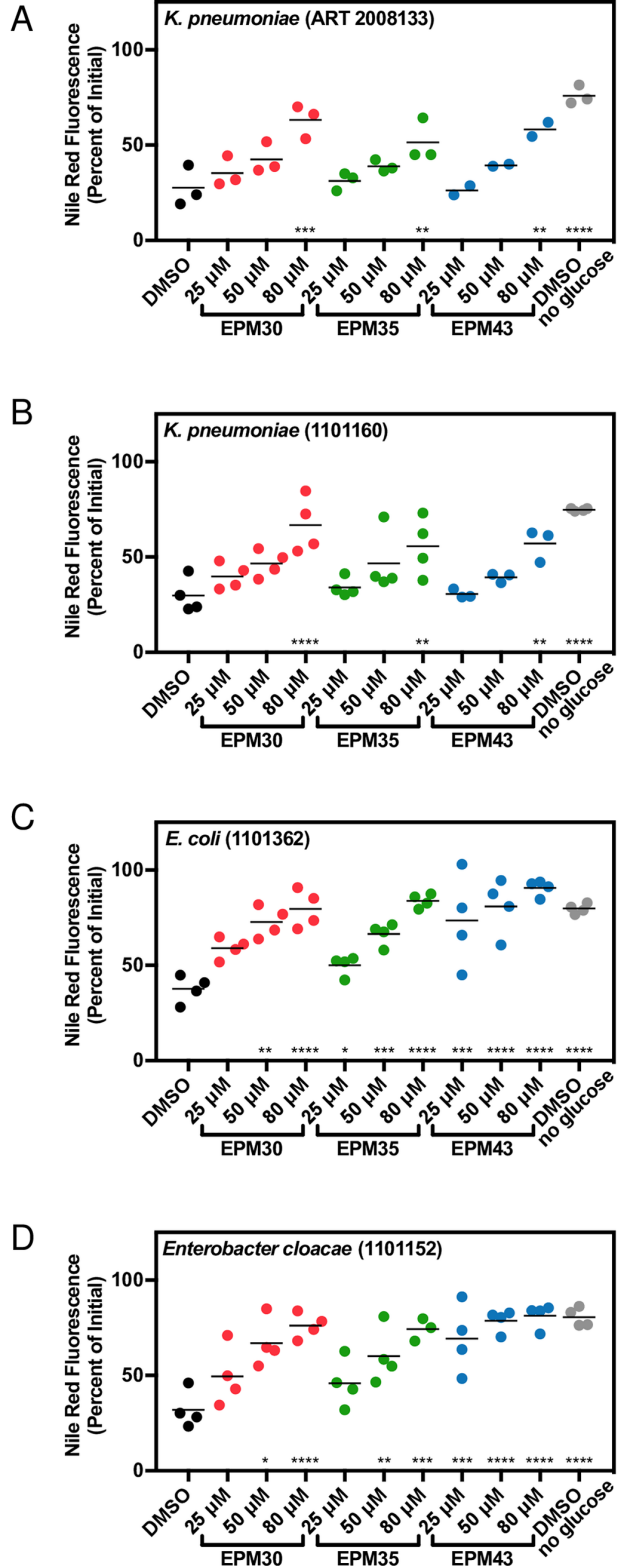
EPM35 and EPM43 block efflux of Nile red from ESKAPE MDR clinical isolates. (A-D) Defined strains obtained from BEI resources were examined for Nile red retention after glucose addition in the presence of the indicated compound. Data for each sample were normalized to the initial fluorescence (100%). Dose response curves at seven minutes after glucose addition. Mean of three biological replicates performed in duplicate. * *p* < 0.05; ** *p* < 0.01; *** *p* < 0.001; ****p < 0.0001 compared to DMSO + glucose by one-way ANOVA and Dunnett’s multiple comparison post-test.

Six pathogens that cause the bulk of MDR nosocomial infections have been dubbed the ESKAPE pathogens: *Enterococcus faecium*, *Staphylococcus aureus*, *Klebsiella pneumoniae*, *Acinetobacter baumannii*, *Pseudomonas aeruginosa*, and *Enterobacter* species [56]. EPM35 and EPM43 significantly reduced Nile red export in MDR clinical isolates of *K*. *pneumoniae* and *Enterobacter cloacae* in addition to *E*. *coli* (**Fig 11**), suggesting these two compounds have biological relevance in MDR strains beyond *Salmonella*.

## Discussion

In-cell screens aimed at identifying chemicals that prevent pathogen intracellular replication have been described. For example screens of FDA-approved drug libraries have uncovered modulators of *Listeria monocytogenes* or *Salmonella enterica* infection [57,58]. Similar screens were performed with macrophages infected with *Mycobacterium tuberculosis* [59] or the yeast *Cryptococcus neoformans* [60]. There are also many reports of high-content screens aimed at identifying genetic disruptors of host-pathogen interactions [61–63]. SAFIRE adopts what we thought would be the most useful aspects of these earlier studies, including a GFP-expressing *Salmonella* to track the microbe, the addition of compounds after infection, automated fluorescence microscopy-based image analysis with MATLAB, and estimation of compound toxicity based on manual visualization of cell morphology and MitoTracker staining. In addition, because the SAFIRE assay includes serum, it avoids compounds that are poor candidates for drug development because of high affinity for serum proteins [64]. These features enabled us to develop SAFIRE as a medium-throughput assay useful for identifying small molecules that interfere with the host-pathogen relationship.

Pathogens require efflux pump activity to survive in host tissues, suggesting modulators of bacterial efflux may be identified with in-cell screens for pathogen survival [30,34]. *Salmonella* encodes nine efflux pumps [65]. The two demonstrated to be required for bacterial survival in cells and in mice are AcrAB-TolC and MacAB [30–33,39]. The first hint that several of the hit compounds may modulate bacterial efflux was the observation that treatment of wild-type *Salmonella* with PAβN or with any of the three EPMs allowed Hoechst to accumulate to higher levels than in vehicle-treated bacteria. We speculate that treatment with PAβN or the EPMs was more effective at raising Hoechst levels than was deletion of the *acrAB*::kan locus because the compounds may target other efflux pumps. We also note that EPMs are not expected to function as clinical antibiotics: EPMs have high MICs in standard broth-based assays [66]. However, EPMs are of interest because of their potential to enhance the activity of existing antibiotics and/or host antimicrobials. These properties further underscore the biology of efflux pumps and highlight the importance of looking beyond MIC assays to identify chemicals with antimicrobial activity under conditions that approximate infection.

All three of our hit compounds bind the efflux pump subunit AcrB, a subunit of the most thoroughly studied RND efflux pump. AcrB integrates into cellular membranes and captures substrates from the outer leaflet of the cytoplasmic membrane or the periplasm [67–69]. The compounds identified bind AcrB more tightly than several known AcrB substrates, such as ethidium (K_D_ of 8.7 +/- 1.9 μM), proflavin (K_D_ of 14.5 +/- 1.1 μM), and ciprofloxacin (K_D_ of 74.1 +/- 2.6 μM) [70]. The chemical structures of the three compounds have some resemblance to known efflux pump inhibitors (**Fig 2E**). EPM30 is a small compound with an aminothiazole core, and several aminothiazole compounds have been identified that inhibit efflux [7,71]. EPM35 is a trifluoro-pyrimidine linked to a piperidine. A very similar compound was suggested to bind the AcrB substrate-binding pocket in an *in silico* screen [72]. EPM43 is a small quinazoline, a planar moiety which is a common drug pharmacophore. Other quinazolines have been identified as inhibitors of bacterial and fungal efflux pumps [73,74]. EPM43 itself has been identified as an inhibitor of fungal dihydrofolate reductase (DHFR), but is not known to inhibit bacterial or human DHFR [75,76]. Where on the AcrB protein the EPMs bind remains unknown. EPM35 and EPM43 potentiate multiple AcrB substrates, suggesting they bind in the hydrophobic trap [77,78]. Alternatively, the EPMs may bind outside of the substrate pocket and, for instance, disrupt AcrB folding, localization or interactions with AcrA. It is notable that the three EPMs do not behave identically in broth assays that monitor export of AcrB substrates, potentiation of antibiotics, or activity against other Gram-negative pathogens, emphasizing that they may not interact identically with AcrB and/or any other molecules they may target.

Why the three EPMs are more potent as antibacterials in mammalian cells than they are as efflux pump inhibitors in broth is not completely clear. A simple model supported by existing data is that the EPMs increase bacterial sensitivity to host AMPs by binding efflux pump subunits, thereby reducing AMP export [79] and decreasing the effective concentration of AMPs. During infection of a whole animal, endogenous AMPs, which are ubiquitous in body fluids, may synergize with EPMs, even in severely immunocompromised patients for whom innate immunity typically remains intact [80,81]. While *Salmonella* RND efflux pumps have not been demonstrated to export AMPs, it is nevertheless encouraging that two of the EPMs inhibit efflux in other major MDR bacterial pathogens, suggesting they may have utility beyond *Salmonella*. Another possible explanation for higher potency during infection than in broth is that the EPMs could accumulate in the SCV, thereby increasing the concentration of compound experienced by the bacterium within host cells. An EPM could also target the host cell and, for instance, increase production of antimicrobial mediators. Alternatively, or in addition, EPMs may interfere with other bacterial processes and/or bind targets that are not present or accessible under the broth conditions tested. To facilitate our understanding of how the EPMs function, it may be useful to identify more potent, less toxic chemical derivatives. Desirable derivatives would have efficacy in SAFIRE, potency in efflux assays, and, most importantly, resensitize MDR pathogens to clinical antibiotics by reducing the antibiotic dosage needed to treat an infection.

The work presented as a whole suggests that SAFIRE enables discovery of chemicals that interfere with the host-pathogen relationship and may have potential as lead compounds for therapeutic development. Advantages of SAFIRE-identified chemicals include that they are unlikely to be inactivated by serum and are fairly non-toxic to mammalian cells. As proof-of-principle, SAFIRE identified not only a traditional antibiotic, chloramphenicol, but also three small molecules with activity against Gram-negative bacterial pathogens. Moreover, because SAFIRE identifies compounds that decrease bacterial load within mammalian cells, the platform may single out chemicals with antibacterial activity that is facilitated by endogenous host antimicrobial peptides, which are broadly distributed across extracellular and intracellular niches. In summary, SAFIRE followed by secondary screening has the potential to identify new and previously overlooked compounds that may be useful as lead compounds for biological and/or antibacterial discovery.

## Materials and methods

### Ethics statement

Animal work was carried out in accordance with the recommendations in the Guide for the Care and Use of Laboratory Animals of the National Institutes of Health. All protocols were approved by the University of Colorado Boulder Institutional Committee for Animal Care and Use (protocol number 2445). Euthanasia was carried out by carbon dioxide asphyxiation followed by cervical dislocation.

### Bacterial strains

The wild-type *S*. *enterica* serovar Typhimurium strain, SL1344, was initially isolated from the blood of an infected calf [26]. For screening and validation in macrophages, SL1344 *sifB*::GFP [20] was grown in Luria-Bertani Broth (LB) with 30 μg/ml streptomycin and 30 μg/ml kanamycin to saturation overnight, diluted to an OD of 0.001 and frozen in 100 μL aliquots in 20% glycerol at -80°C. Prior to infection, aliquots were grown in 5 mL cultures of LB with 30 μg/ml streptomycin and 30 μg/ml kanamycin for 18 hours at 37°C with aeration. Bacterial strains were routinely grown in LB with antibiotics (**Table 1**): 30 μg/ml streptomycin, 30 μg/ml kanamycin, 50 μg/ml ampicillin, 10 μg/ml tetracycline, and/or 1.15 μg/ml meropenem. The *acrAB*::*kan* and *macAB*::*kan* strains were constructed as described [82]. *S*. *enterica* subsp. *enterica*, serovar Typhimurium strain S10801, NR-22067 is a multidrug resistant isolate from a calf with sepsis [83]. This strain and others as indicated (**Table 1**) were obtained through BEI resources, NIAID, NIH.

### Cell culture

Murine macrophage-like RAW 264.7 cells and HeLa human epithelial cells were obtained from the American Type Tissue Collection. BMDMs were isolated as previously described [21]. Briefly, marrow was flushed from the femurs of 1- to 4-month-old 129SvEvTac mice (Taconic Laboratories) bred in-house. Mononuclear cells were separated using Histopaque-1083 (Sigma), washed, and directly seeded into assay plates at 1 x 10^5^ cells/ml in complete medium supplemented with 35% conditioned media from 3T3 cells expressing MCSF. Media were refreshed three days later. After 1 week, media were replaced with 100 μL fresh media and cells were infected as described below. All three types of cells were grown in DMEM high glucose (Sigma) supplemented with 10% fetal bovine serum, 2 mM L-glutamine, 1 mM sodium pyruvate, 10 mM HEPES, and 50 μM β-mercaptoethanol. Cells were maintained in a 5% CO_2_ humidified atmosphere at 37°C. For screening, frozen aliquots of RAW 264.7 were thawed and allowed to expand for three days prior to seeding; other experiments were performed with cultures between passages four and 20.

### Bacterial infections for SAFIRE and CFU plating

*SAFIRE—*RAW 264.7 macrophages (7 x 10^3^ in 40 μL or 5 x 10^4^ in 100 μL) were seeded, respectively, in 384- or 96-well black-walled glass-bottomed plates (Brooks Automation). Twenty-four hours post-seeding, bacteria in 20 or 50 μL PBS were added to a final concentration of 1 x 10^7^ CFU/mL, conditions yielding infection of approximately 70% of macrophages at 18 hours post-infection with minimal macrophage toxicity. The *sifB*::GFP bacterial reporter strain was used to minimize green signal from extracellular bacteria. Forty-five minutes after bacterial addition, 20 or 50 μL gentamicin was added to a final concentration of 40 μg/mL, which did not affect intracellular infection but inhibited replication of extracellular bacteria. At two hours post-infection, 200 or 500 nL compound was added using a pin tool (CyBio) to yield a final concentration of 25 μM. Each assay plate included rifampicin and DMSO controls. In some experiments, media were removed and replaced with fresh media containing 40 μg/mL gentamicin and the indicated concentrations of drugs. At 17.5 hours post-infection, PBS containing MitoTracker Red CMXRos (Life Technologies) was added to a final concentration of 300 nM or 100 nM, for 384- or 96-well, respectively. Thirty minutes later, 16% paraformaldehyde was added to a final concentration of 1–2% and incubated at room temperature for 15 minutes. Wells were washed twice with PBS and stained for 20 minutes with 1 μM DAPI; wells were washed twice and stored in 90% glycerol in PBS until imaging. The Z’-factor of the screening platform was 0.59 and 0.48 in 96-well and 384-well plates, respectively, within published ranges for complicated cell-based screens [22,60–62].

*HeLa cells—*Infections of HeLa cells with *Salmonella* were performed as above except that 1 x 10^4^ cells were seeded, and cells were infected with *Salmonella* constitutively expressing GFP from the *rpsM* locus because *sifB*::GFP is poorly expressed in HeLa cells. In addition, plates were spun for five minutes at 500 x *g* after addition of bacteria to enhance infection.

*CFU—*Infections were performed as described above, except cells were seeded in 96-well tissue culture coated plates (Greiner). At 18 hours post-infection, wells were washed three times in PBS, lysed with 30 μL 0.1% Triton X-100, diluted and plated to determine CFU.

### Image acquisition, MATLAB-based screening analysis, and hit selection

High magnification images were acquired on an Olympus IX81 inverted widefield microscope (40X) or CV1000 spinning disk confocal microscope (20X). For screening, three-color images were acquired at 10X on a Cellomics ArrayScan VTI (Thermo) and exported to DIB files. At least two fields were imaged per well for all experiments, and compounds were screened in duplicate. To quantify intracellular bacterial load we developed an automated MATLAB script (“SAFIRE_ArrayScan”, “SAFIRE_OlympusIX81” and “SAFIRE_CV1000” on MATLAB File Exchange, https://www.mathworks.com/matlabcentral/fileexchange/). Briefly, the algorithm identifies macrophage borders via watershed segmentation using DAPI and MitoTracker. To identify bacteria, the user supplies an empirically determined GFP threshold that maximizes signal to noise based on uninfected and untreated controls. Within each macrophage, the number of pixels above the GFP threshold is counted. If more than two pixels are above the GFP threshold, the macrophage is labeled infected. The script calculates the percentage of macrophages infected in the image. Raw data for at least two images from the same well are averaged to yield one value for each well. Raw screening data were subjected to B-score normalization because we identified significant row and column effects by a method previously described [22]. To determine significance, we employed the modified one-sample t-test [23] by fitting the variances of replicates to an inverse gamma distribution [25]. The micrographs of the 461 preliminary positives were examined by the human eye to eliminate compounds that clearly destroyed the macrophages (host toxic). Assay positives were defined as having a *p*-value less than 0.05 and a B-score one standard deviation below the mean.

### Broth antibacterial activity assays

Overnight *Salmonella* cultures were washed three times in PBS and diluted to an OD_600_ of 0.01 in MHB in 96-well flat-bottom plates. Compound was added using a pin tool (CyBio) or manually, yielding a final concentration of no more than 1% DMSO. Plates were grown at 37°C shaking and OD_600_ was monitored using a BioTek Eon or Synergy H1 incubator shaker microplate absorbance reader. MICs were defined as the concentration at which no growth was visually observed. The Fractional Inhibitory Concentration Index (FICI) was calculated for wells showing no visible growth (FICI = [agent A] / [MIC agent A] + [agent B] / [MIC agent B]) [84]. For experiments with polymyxin B, bacteria were grown in M9 minimal media supplemented with 100 mM Tris pH 7.4, 0.35% glycerol, 0.002% histidine, 10 mM MgCl_2_, and 0.1% casamino acids and 5 μg/mL polymyxin B. For experiments with LL37, bacteria were grown in M9 minimal media supplemented with 0.4% dextrose, 0.004% histidine, 1 mM MgSO_4_, and 5 μg/ml LL37.

### Hoechst assays

Hoechst accumulation assays were performed essentially as described [35]. Briefly, overnight *Salmonella* cultures were washed three times in PBS and diluted to an OD_600_ of 0.1 in PBS with 2.5 μM Hoechst 33342 in the presence of the indicated concentrations of compounds. Fluorescence was monitored on a Biotek Synergy 2 with a 360/40 nm excitation filter and 460/40 nm emission filter. The maximum Hoechst fluorescence over 60 minutes of incubation was normalized to the signal from the equivalent number of heat-killed bacteria, after subtraction of autofluorescence signal determined from compound incubated in the absence of bacteria. EC50s were determined using a 4-parameter nonlinear fit constrained using DMSO-treated Wild-Type as the minimum and Heat Killed as the maximum (GraphPad Prism). Concentrations of PAβN used in all assays were determined by titration with the *Salmonella* wild-type strain in the corresponding assay.

### Nile red and ethidium bromide assays

Nile red assays were adapted from an established protocol [50]. Briefly, overnight *Salmonella* LB cultures were washed in PBS with 1 mM MgCl_2_ and resuspended at an OD_600_ of 2.0. Cells were incubated in 10 μM Nile red for three hours at 37°C in glass tubes on a roller drum and then at room temperature standing for one hour. Cells were pelleted at 2,050 x *g*, resuspended in PBS with 1 mM MgCl_2_, and 200 μl was added to 96-well black walled plates (Greiner) with compound at the indicated concentrations. In washout experiments, after 35 minutes of incubation with compound, cells were centrifuged at 16,000 x *g*, resuspended in PBS with 1mM MgCl_2_ without compound and aliquoted into 96-well black walled plates (Greiner). During loading into plates (~ 20 minutes), bacteria effluxed some Nile red even in the absence of glucose (**S4 Fig**). Samples were read using a Varioskan Flash Multimode Reader at 540 nm (excitation) and 625 nm (emission) or a Biotek Synergy H1 at 560 nm and 655 nm. To activate efflux, glucose was added to a final concentration of 2 mM.

Ethidium bromide (EtBr) assays were performed similarly to Nile red assays with the following changes. Cells were incubated with 10 μM of carbonyl cyanide m-chlorophenylhydrazone (CCCP) for 15 minutes, then incubated with 10 μM of EtBr for one hour at 37°C with aeration, pelleted at 2,050 x *g*, aliquoted and combined with compound at the indicated concentrations in 96-well black walled plates (Greiner). Plates were monitored with a Biotek Synergy H1 at 510 nm and 600 nm.

### Nitrocefin hydrolysis assays

Bacteria were subcultured to mid-log phase, washed, and combined with 100 μM nitrocefin and the indicated concentrations of drugs in 96-well plates in 200 μL [41]. Washes and incubations were performed in 20 mM KPO_4_, pH 7.0, 1 mM MgCl_2_. Absorbance (486 nm) was measured on a BioTek Eon or Synergy H1 spectrophotometer every 60 seconds for one hour. To observe efflux inhibition, *E*. *coli* RAM121 [28] were added to plates at a final OD_600_ of 10. This strain produces an OmpC variant with a larger pore size to allow increased influx of nitrocefin and other bulky molecules, and nitrocefin is hydrolyzed by the endogenous AmpC beta-lactamase. To measure outer membrane permeability, wild-type *Salmonella* harboring beta-lactamase *(bla)*-expressing pACYC177-mTagBFP2 [29] were added to plates at a final OD_600_ of 0.1.

### Tetramethylrhodamine methyl ester (TMRM) permeability assays

Wild-type *Salmonella* harboring pACYC177-mTagBFP2 [29] were subcultured to mid-log phase, diluted to 1 x 10^6^ CFU/ml in PBS, aliquoted into flow cytometry tubes, and treated with the indicated concentrations of compounds. TMRM was immediately added to a final concentration of 100 nM. After incubation for 30 minutes at 37°C, samples were analyzed on a CyAn ADP (Beckman Coulter) in channels FL6 and FL2. Data were analyzed using FlowJo; bacteria were gated based on side scatter and BFP signal in the FL6 channel, and the FL2 median fluorescence intensity (MFI) was calculated.

### Bacterial swimming membrane permeability assays

Saturated overnight cultures were diluted to an OD_600_ of 0.01 in LB, and 1 μL was injected into the center of low (0.25%) agar LB plates. Ten microliters of the indicated compounds up to concentrations of 100 mM were added to sterilized Whatman paper disks (diameter 0.7 cm) placed equidistant from the plate center; solubility issues arose at concentrations above 100 mM. Plates were incubated face up at 37°C overnight; no change in halo size was observed between 14–24 hours incubation. Plates were imaged using a Gel Logic 200 imaging system, and halo radius (distance from center of disk to outermost edge of halo) was measured using ImageJ.

### Isothermal titration calorimetry (ITC)

ITC was used to examine the binding of EPMs to the purified AcrB transporter. Measurements were performed on a Microcal iTC200 (Northampton, MA) at 25°C. Before titration, the protein was thoroughly dialyzed against buffer containing 20 mM Na-HEPES (pH 7.5), 0.05% n-dodecyl-μ-maltoside (DDM) and 5% dimethyl sulfoxide (DMSO). The protein concentration was determined using the Bradford assay. The protein sample was then adjusted to a final monomeric concentration of 10 μM. Ligand solution consisting of 100 μM EPM30, EPM35 or EPM43 in 20 mM Na-HEPES (pH 7.5), 0.05% DDM and 5% DMSO was prepared as the titrant. The protein and ligand samples were degassed before they were loaded into the cell and syringe. Binding experiments were carried out with the protein solution (0.2 ml) in the cell and the ligand as the injectant. Two microliter injections of the ligand solution were used for data collection.

Injections occurred at intervals of 60 s, and the duration time of each injection was 4 s. Heat transfer (μcal/s) was measured as a function of elapsed time (s). The mean enthalpies measured from injection of the ligand in the buffer were subtracted from raw titration data before data analysis with ORIGIN software (MicroCal). Titration curves were fitted by a nonlinear least-squares method to a function for the binding of a ligand to a macromolecule. Nonlinear regression fitting to the binding isotherm provided us with the equilibrium binding constant (*K*_*A*_ = 1/*K*_*D*_) and enthalpy of binding (*ΔH*). Based on the values of *K*_*A*_, the change in free energy (*ΔG*) and entropy (*ΔS*) were calculated with the equation: *ΔG* = —*RT* ln*K*_*A*_ = *ΔH*—*TΔS*, where *T* is 273 K and *R* is 1.9872 cal/K per mol. Calorimetry trials were also carried out in the absence of AcrB using the same experimental conditions. No change in heat was observed in the injections throughout the experiment.

### Statistics

Statistical tests were applied as indicated in figure legends using GraphPad/Prism and/or JMP software.

## Supporting information

S1 Table SAFIRE screening results. (XLSX)

S1 Fig Comparison of compound activity [25 μM] in CFU assays versus SAFIRE. The top 75 compounds as determined by SAFIRE (96-well format) are shown (purple circles). EPM30 (red triangle); EPM35 (green square); EPM43 (blue circle); chloramphenicol (gray hexagon). For comparison, antibiotic controls rifampicin, ampicillin, and ciprofloxacin are indicated with arrows (open circles).
(TIF)

S2 Fig The EPMs were not potentiated by gentamicin in broth. Bacterial growth in MHB across a range of gentamicin concentrations was not significantly altered by the presence of an EPM, as indicated by Fractional Inhibitory Concentrations Indices (FICI) of greater than 0.5.
(TIF)

S3 Fig EPMs reduced efflux of ethidium bromide and Nile red. A) Ethidium bromide, B) Nile red, C) Nile red with compounds washout. Mean and SD from representative efflux assays performed twice in duplicate. Upper dotted lines, initial fluorescence for DMSO-treated cells without glucose; lower dotted lines, endpoint fluorescence for DMSO-treated cells exposed to glucose. * *p* < 0.05; ** *p* < 0.01 determined by t-test of slopes calculated from linear fit of 0–10 minutes (EtBr) or 0–2 minutes (Nile red) relative to buffer.
(TIF)

S4 Fig DMSO-treated cells efflux Nile red in the absence of glucose. (A) Nile red-loaded bacteria were washed, combined with the indicated concentrations of compounds and fluorescence was immediately measured. Data shown are mean + SD. These data suggest that the discrepancies in starting fluorescence (S4B Fig) are due to the time between compound addition and the beginning of measurement (15–20 minutes). As indicated here, during this timeframe DMSO-treated cells efflux the dye even in the absence of glucose. Thus, EPM35, EPM43, and PAβN inhibit basal loss of Nile red, but treatment with EPM30 led to an immediate reduction in fluorescence. (B) Bacteria remain intact and viable after 20 minutes incubation in 75 μM EPM30, indicating the immediate reduction in fluorescence in (A) is not due to death of the bacteria. It is possible that EPM30 reduces Nile red fluorescence by quenching or by altering membrane properties, as Nile red fluorescence is highly dependent on membrane polarity, content, and dynamics.
(TIF)

S5 Fig EPMs did not disrupt bacterial swimming. Disk diffusion assay. Bacteria were injected into the center of the plate (*); 10 μl of the indicated compound (top) or vehicle was spotted onto filter paper disks. Sixteen hours later, plates were imaged. Representative images from one of three independent experiments.
(TIF)

S6 Fig EPM30 interacts with the efflux pump AcrB. Representative ITC for the binding of EPM30 to *E*. *coli* AcrB. Each peak in the upper graph corresponds to the injection of 2 μL of 100 μM of the EPM in buffer containing 20 mM Na-HEPES (pH 7.5), 0.05% DDM and 5% DMSO into the reaction containing 10 μM of E. coli monomeric AcrB in buffer containing 20 mM Na-HEPES (pH 7.5), 0.05% DDM and 5% DMSO. The lower graph shows the cumulative heat of reaction displayed as a function of injection number. The solid line is the least-square fit to the experimental data.
(TIF)

S7 Fig Polymyxin B [5 μg/mL] did not increase Hoechst accumulation or Nile red retention in the presence of EPMs. (A, top) Hoechst accumulation quantitated as in Fig 2. (A, bottom) The DMSO, no-polymyxin B-treated samples were subtracted from treated samples (gray bars). Assuming additivity as the null hypothesis, the sum of the 5 μg/ml polymyxin B sample and each EPM sample was calculated (white bars). No significant differences were identified between observed and calculated data, suggesting that EPMs and polymyxin B do not synergize in this assay. (B) Nile red efflux quantitated as in Fig 4 and analysis performed as in A. * *p* < 0.05, ** *p* < 0.01, *** *p* < 0.001, **** *p* < 0.0001 calculated using one-way ANOVA with Dunnett’s post-test.
(TIF)
