# Supplementary figures and images for: A cell-based infection assay identifies efflux pump modulators that reduce bacterial intracellular load
Source: PLoS Pathog. 2018 Jun 7;14(6):e1007115. doi: 10.1371/journal.ppat.1007115 (PMC6007937; doi:10.1371/journal.ppat.1007115)

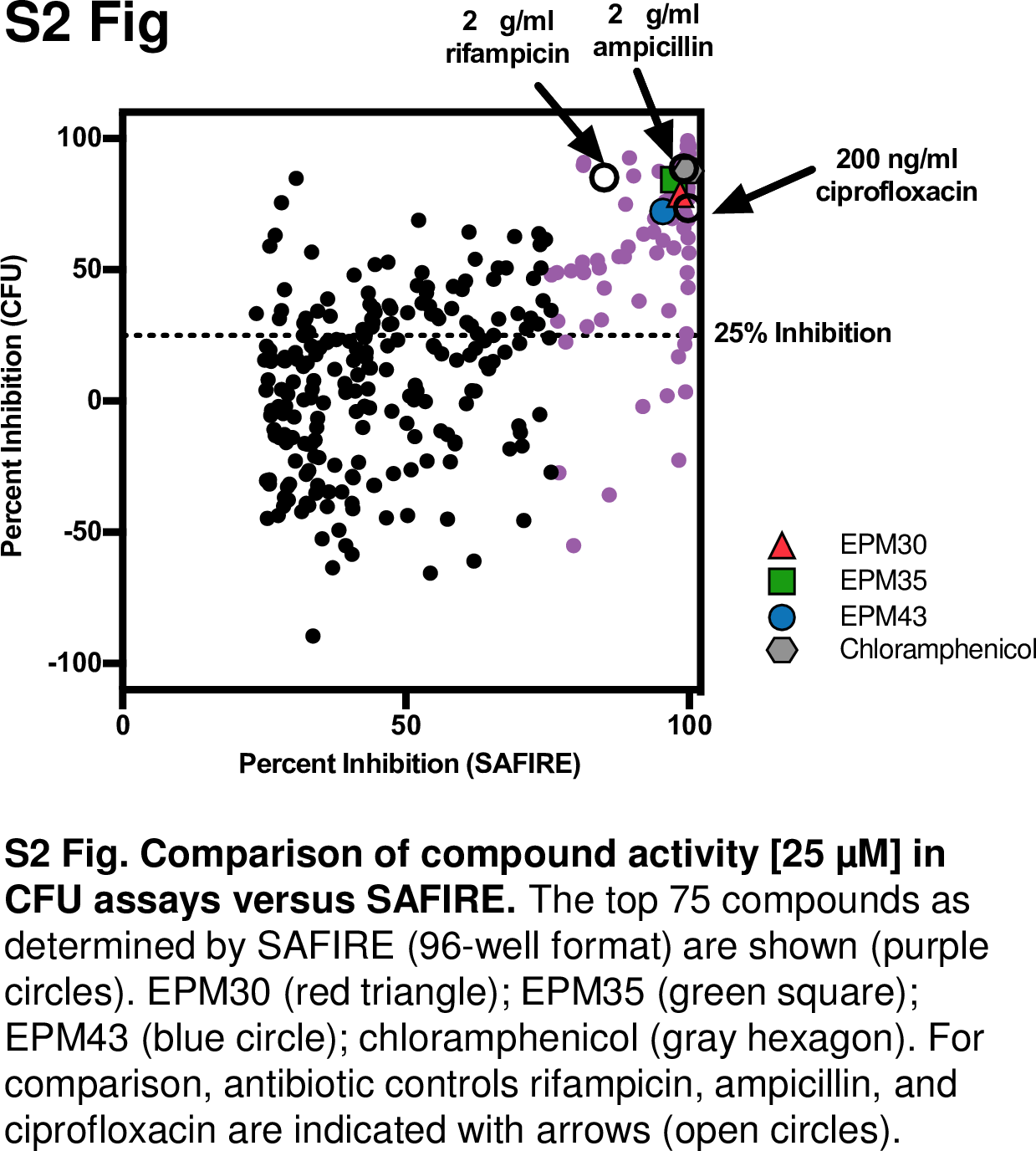

Supplement: S1 Fig — The top 75 compounds as determined by SAFIRE (96-well format) are shown (purple circles). EPM30 (red triangle); EPM35 (green square); EPM43 (blue circle); chloramphenicol (gray hexagon). For comparison, antibiotic controls rifampicin, ampicillin, and ciprofloxacin are indicated with arrows (open circles). (TIF) [file ppat.1007115.s002.tif]

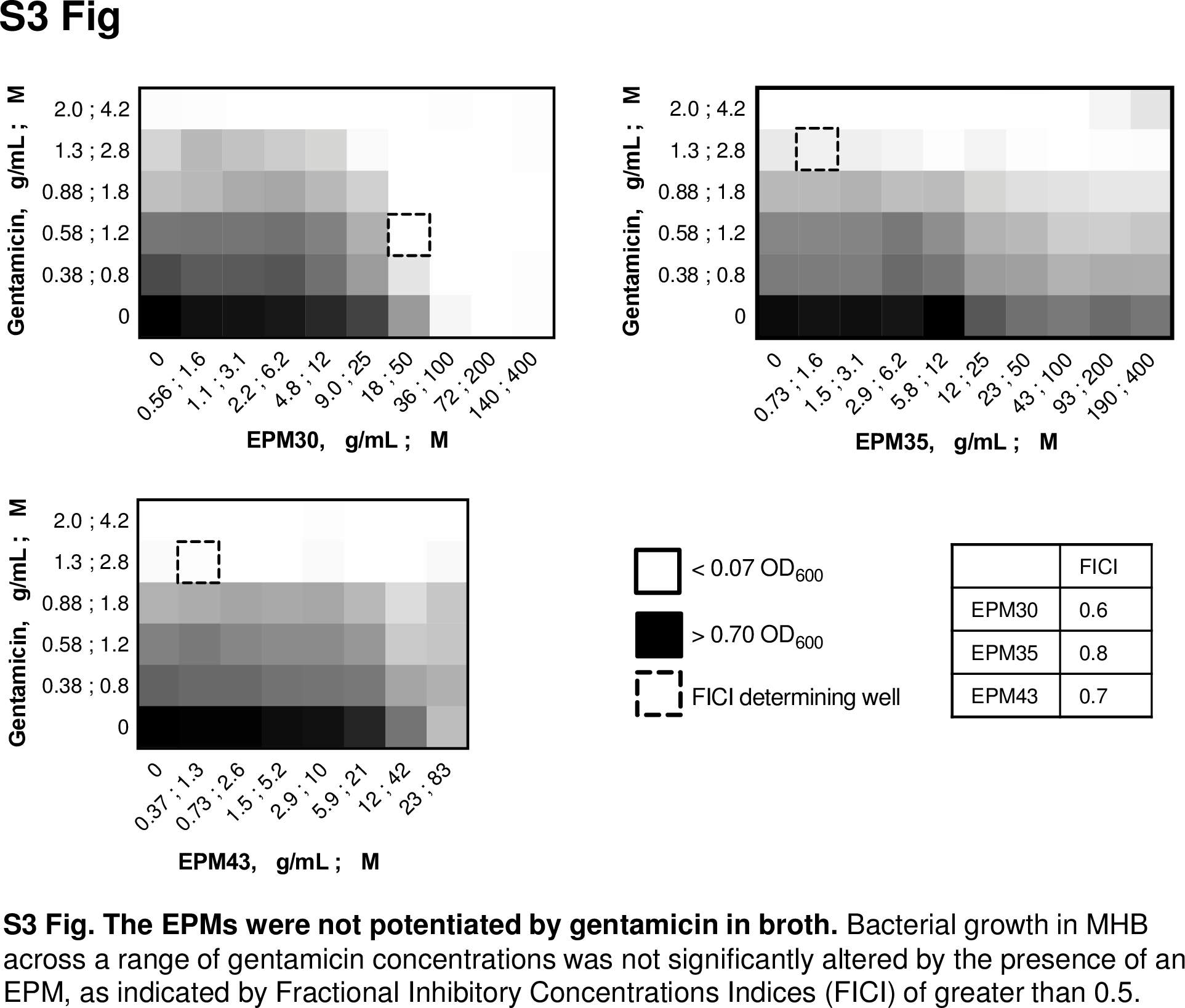

Supplement: S2 Fig — Bacterial growth in MHB across a range of gentamicin concentrations was not significantly altered by the presence of an EPM, as indicated by Fractional Inhibitory Concentrations Indices (FICI) of greater than 0.5. (TIF) [file ppat.1007115.s003.tif]

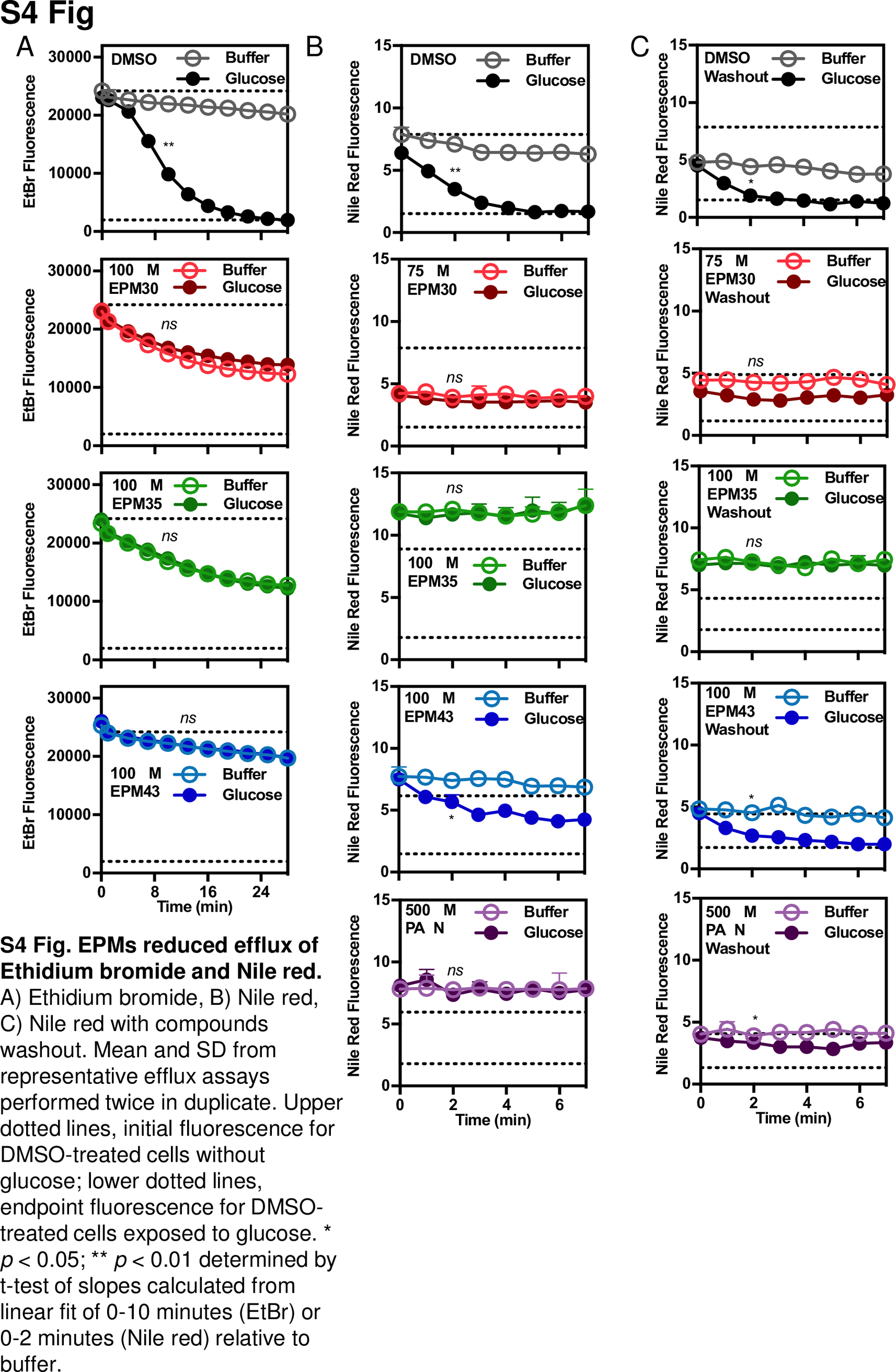

Supplement: S3 Fig — A) Ethidium bromide, B) Nile red, C) Nile red with compounds washout. Mean and SD from representative efflux assays performed twice in duplicate. Upper dotted lines, initial fluorescence for DMSO-treated cells without glucose; lower dotted lines, endpoint fluorescence for DMSO-treated cells exposed to glucose. * p < 0.05; ** p < 0.01 determined by t-test of slopes calculated from linear fit of 0–10 minutes (EtBr) or 0–2 minutes (Nile red) relative to buffer. (TIF) [file ppat.1007115.s004.tif]

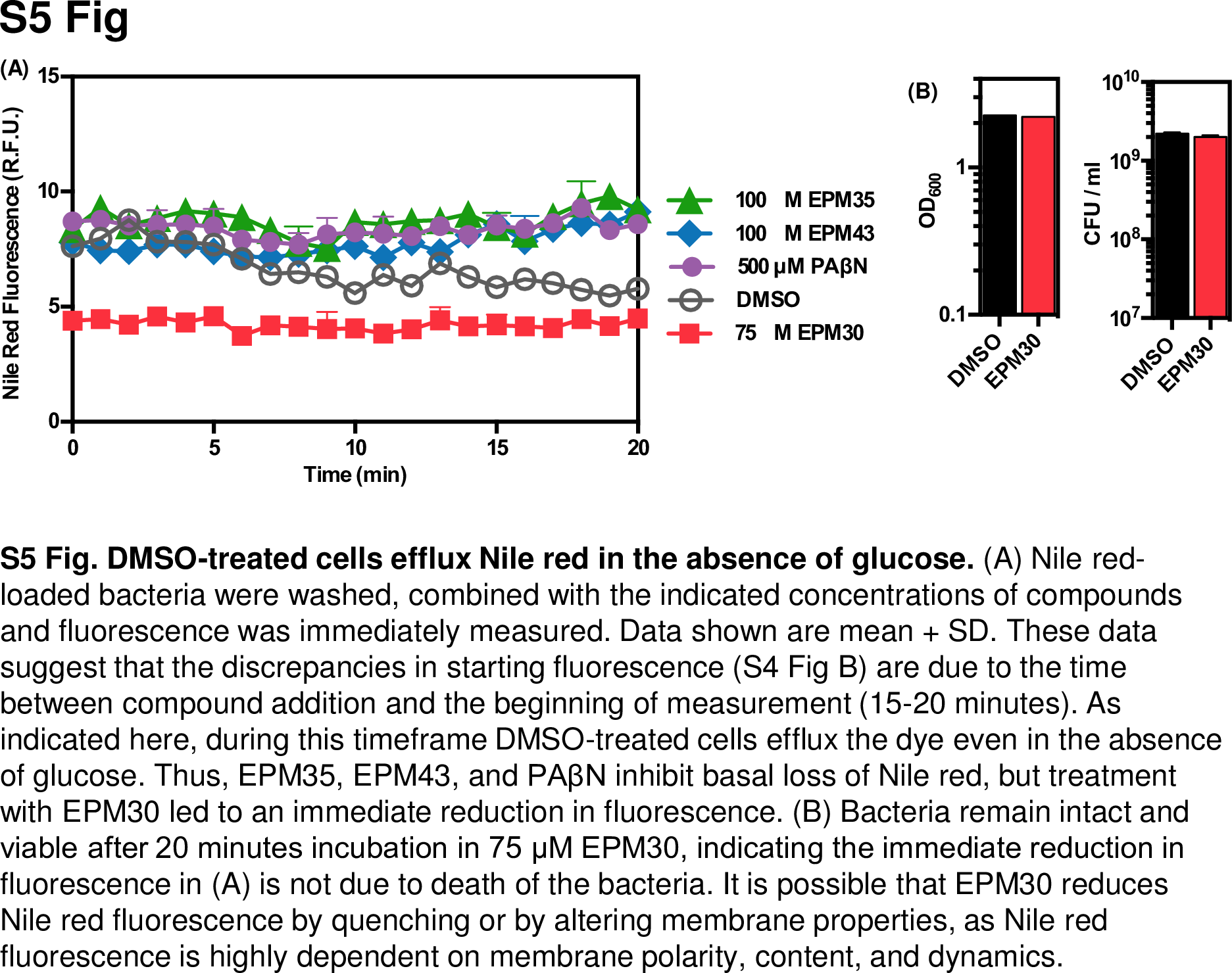

Supplement: S4 Fig — (A) Nile red-loaded bacteria were washed, combined with the indicated concentrations of compounds and fluorescence was immediately measured. Data shown are mean + SD. These data suggest that the discrepancies in starting fluorescence (S4B Fig) are due to the time between compound addition and the beginning of measurement (15–20 minutes). As indicated here, during this timeframe DMSO-treated cells efflux the dye even in the absence of glucose. Thus, EPM35, EPM43, and PAβN inhibit basal loss of Nile red, but treatment with EPM30 led to an immediate reduction in fluorescence. (B) Bacteria remain intact and viable after 20 minutes incubation in 75 μM EPM30, indicating the immediate reduction in fluorescence in (A) is not due to death of the bacteria. It is possible that EPM30 reduces Nile red fluorescence by quenching or by altering membrane properties, as Nile red fluorescence is highly dependent on membrane polarity, content, and dynamics. (TIF) [file ppat.1007115.s005.tif]

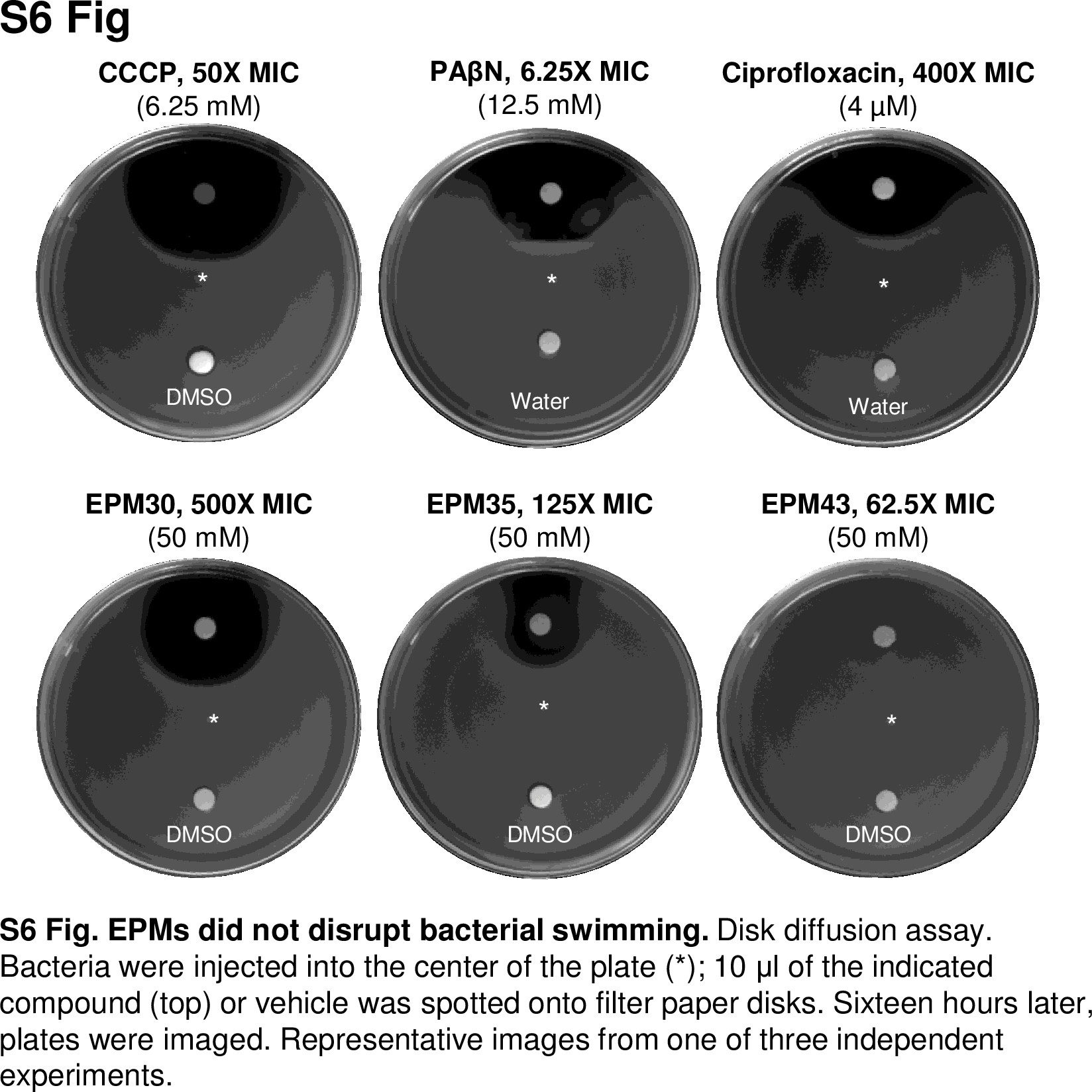

Supplement: S5 Fig — Disk diffusion assay. Bacteria were injected into the center of the plate (*); 10 μl of the indicated compound (top) or vehicle was spotted onto filter paper disks. Sixteen hours later, plates were imaged. Representative images from one of three independent experiments. (TIF) [file ppat.1007115.s006.tif]

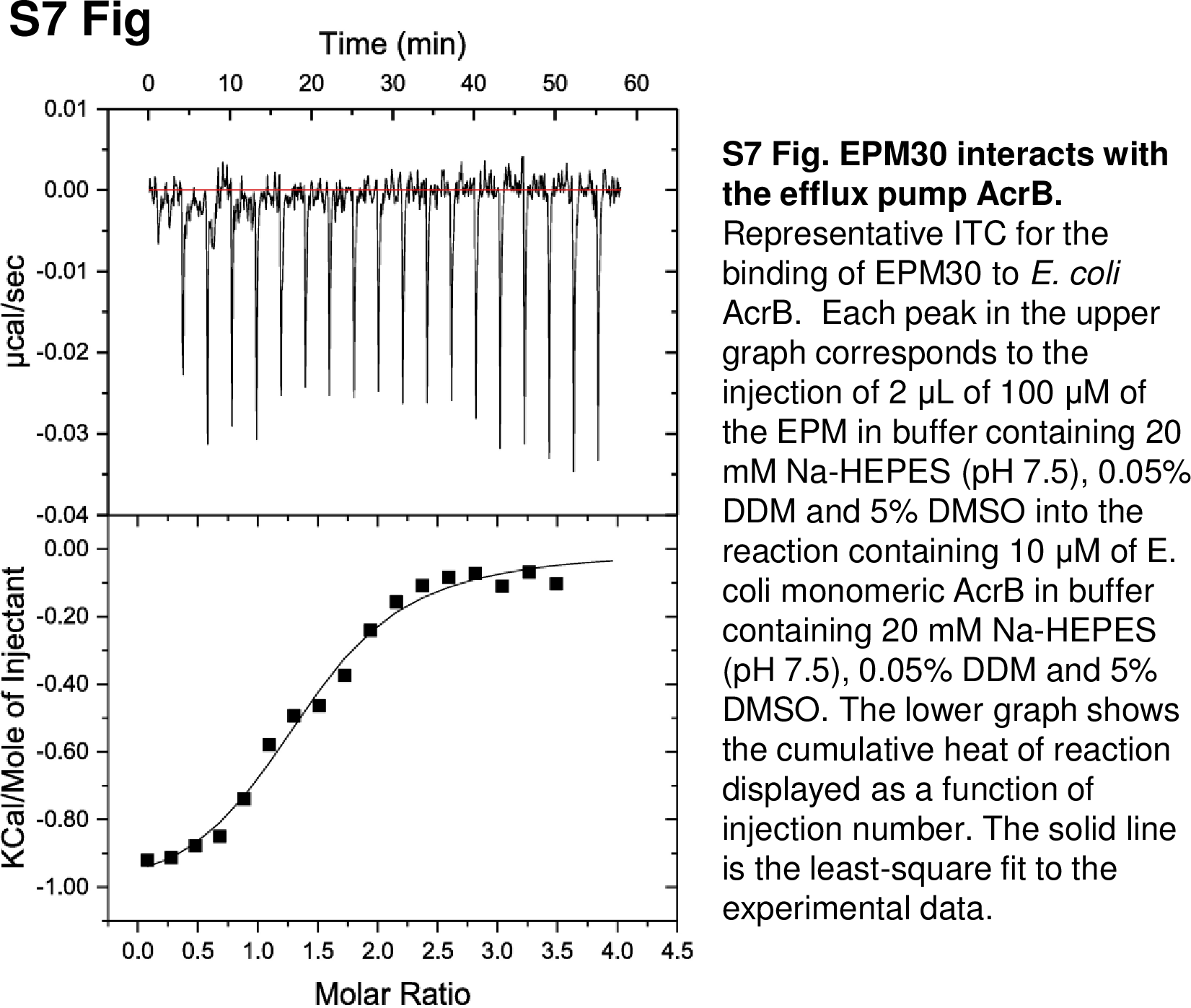

Supplement: S6 Fig — Representative ITC for the binding of EPM30 to E. coli AcrB. Each peak in the upper graph corresponds to the injection of 2 μL of 100 μM of the EPM in buffer containing 20 mM Na-HEPES (pH 7.5), 0.05% DDM and 5% DMSO into the reaction containing 10 μM of E. coli monomeric AcrB in buffer containing 20 mM Na-HEPES (pH 7.5), 0.05% DDM and 5% DMSO. The lower graph shows the cumulative heat of reaction displayed as a function of injection number. The solid line is the least-square fit to the experimental data. (TIF) [file ppat.1007115.s007.tif]

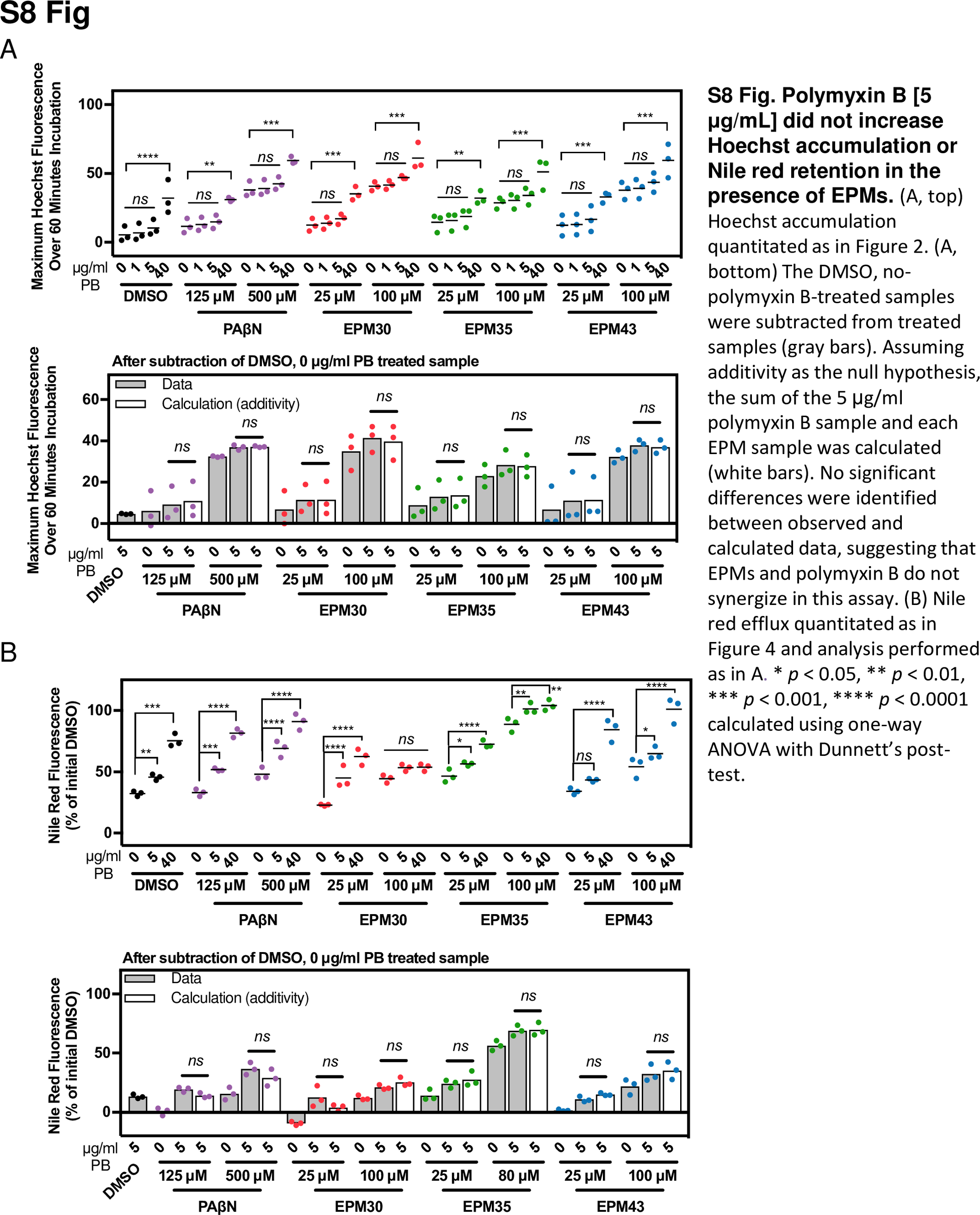

Supplement: S7 Fig — (A, top) Hoechst accumulation quantitated as in Fig 2. (A, bottom) The DMSO, no-polymyxin B-treated samples were subtracted from treated samples (gray bars). Assuming additivity as the null hypothesis, the sum of the 5 μg/ml polymyxin B sample and each EPM sample was calculated (white bars). No significant differences were identified between observed and calculated data, suggesting that EPMs and polymyxin B do not synergize in this assay. (B) Nile red efflux quantitated as in Fig 4 and analysis performed as in A. * p < 0.05, ** p < 0.01, *** p < 0.001, **** p < 0.0001 calculated using one-way ANOVA with Dunnett’s post-test. (TIF) [file ppat.1007115.s008.tif]
